# Cyclodextrin-Enabled Enantioselective Complexation Study of Cathinone Analogs

**DOI:** 10.3390/molecules29040876

**Published:** 2024-02-16

**Authors:** András Dohárszky, Eszter Kalydi, Gergely Völgyi, Szabolcs Béni, Ida Fejős

**Affiliations:** 1 Department of Pharmacognosy, Semmelweis University, Üllői út 26, H-1085 Budapest, Hungary; doharszky.andras@stud.semmelweis.hu (A.D.); kalydi.eszter@semmelweis.hu (E.K.); 2 Department of Organic Chemistry, Semmelweis University, Hőgyes Endre utca 7, H-1092 Budapest, Hungary; 3 Department of Pharmaceutical Chemistry, Semmelweis University, Hőgyes Endre utca 7, H-1092 Budapest, Hungary; volgyi.gergely@egis.hu; 4 Department of Analytical Chemistry, Institute of Chemistry, ELTE Eötvös Loránd University, Pázmány Péter sétány 1/A, H-1117 Budapest, Hungary

**Keywords:** enantioseparation, complex stability constant, enantioselectivity, chiral capillary electrophoresis, affinity capillary electrophoresis, cathinone derivatives, cathinone analogs, ROESY NMR, Job’s plot, CE-pH titration, acid dissociation constant

## Abstract

The characteristic alkaloid component of the leaves of the catnip shrub (*Catha edulis*) is cathinone, and its synthetic analogs form a major group of recreational drugs. Cathinone derivatives are chiral compounds. In the literature, several chiral methods using cyclodextrins (CDs) have been achieved so far for diverse sets of analogs; however, a comprehensive investigation of the stability of their CD complexes has not been performed yet. To characterize the enantioselective complex formation, a systematic experimental design was developed in which a total number of 40 neutral, positively, and negatively charged CD derivatives were screened by affinity capillary electrophoresis and compared according to their cavity size, substituent type, and location. The functional groups responsible for the favorable interactions were identified in the case of para-substituted cathinone analog mephedrone, flephedrone, and 4-methylethcathinone (4-MEC) and in the case of 3,4-methylendioxy derivative butylone and methylenedioxypyrovalerone (MDPV). The succinylated-β-CD and subetadex exhibited the highest complex stabilities among the studied drugs. The complex stoichiometry was determined using the Job’s plot method, and the complex structures were further studied using ROESY NMR measurements. The results of our enantioselective complex formation study can facilitate chiral method development and may lead to evaluate potential CD-based antidotes for cathinone analogs.

## 1. Introduction

Khat (*Catha edulis* (Vahl) Forssk. ex Endl., Celestraceae) is a slow-growing shrub native to East Africa and the Arabian Peninsula. Traditionally, its leaves have been chewed for their psychostimulant effects, with a history spanning over a thousand years in Yemen and Ethiopia. Khat-chewing significantly influences daily life, playing a role in cultural celebrations and political assemblies. The key alkaloid in khat leaves is cathinone, which induces central nervous system stimulation, resulting in effects such as excitation, euphoria, appetite suppression, hyperventilation, hyperthermia, analgesia, and heightened sensation. Notably, these effects bear similarities to those observed during amphetamine consumption, possibly owing to structural resemblances between the compounds.

**Figure 1:**
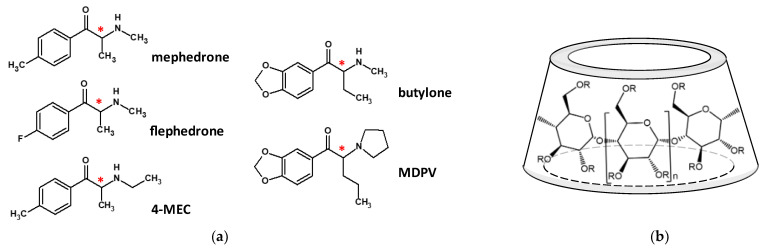
(**a**) Chemical structure of the studied cathinone analogs; the 3 para-substituted analogs: mephedrone (4-methylmethcathinone), flephedrone (4-fluoromethcathinone), and 4-methylethcathinone (4-MEC) and the 2 methylenedioxy analogs: butylone and 3,4-methylenedioxypyrovalerone (MDPV). The chiral centers are marked with an asterisk. (**b**) Schematic structure of the cyclodextrins (R stands for substituents).

The synthetic derivatives of cathinone form a major group of (designer) recreational drugs. The most common source of designer drugs is the internet, where they are often marketed as “not for human consumption”, horticultural chemicals, fumigants, or research substances [1]. The cathinone analogs possess diverse substitution patterns, but the general backbone is the 2-amino-1-phenylpropan-1-one core (Figure 1a). Typically, the phenyl group is substituted with fluoro, alkyl, or alkoxy groups at positions 2, 3, and/or 4, the propyl side chain may be extended, and the amino group may be secondary or tertiary via the attached alkyl substituent(s). The methylenedioxy substitution converts the psychostimulant effect into a psychotropic effect. The number and consumption of cathinone analog drugs are growing rapidly, posing a challenge for regulations to keep pace. The health risks associated with their recreational use are not negligible, not only because they are addictive and harmful to health but also because they may cause unexpected effects. Moreover, the healthcare system is often unprepared to handle acute conditions resulting from irresponsible use and overdose. In such cases, having compounds and preparations available that can rapidly eliminate these substances from the body would be beneficial.

Besides other supramolecular macrocycles, cyclodextrins (CDs) may be suitable for this purpose due to their excellent encapsulation properties. CDs are cyclic oligosaccharides with α-(1,4)-linked *D*-glucopyranose units on the surface of the truncated cone (Figure 1b). The polar nature of the outer surface of CDs ensures their good water solubility, while their weakly hydrophobic inner cavity can accommodate more apolar guest molecules via inclusion complex formation. Due to their excellent biocompatibility, stability, and solubility-enhancing activity, CDs are the most widely applied excipients in pharmaceutics. However, chemically designed CDs may bind with toxic/active substances thus reversal effects could be achieved [2,3]. This is clearly demonstrated by the supramolecular antidote CD derivative, sugammadex (Bridion^®^), which is used to immediately suspend the effects of several neuromuscular blockers (e.g., rocuronium, vecuronium) due to a high selectivity encapsulation with outstanding stability.

The versatile complexation properties of CDs could also be utilized in analytics. As CDs possess various chiral centers, their inner cavity could be considered a chiral microenvironment. Nowadays, the enantioselective analysis of chiral substances is essential. CDs can effectively recognize and separate the enantiomers; thus, they are widely used chiral selectors. However, the development of a chiral separation method is not easily predictable; their success as chiral selectors could be fine-tuned due to the broad range of CD derivatives.

Cathinone derivatives are chiral compounds; in the literature, several chiral method developments using CDs have been carried out so far for diverse sets of cathinone derivatives, among which the majority is capillary electrophoretic (CE) methods of solid samples [4,5,6,7,8,9,10,11,12,13,14,15], but methods from urine [16] or hair samples [17] are also present, and an RP-HPLC method using sulfated-β-CD as chiral selector added to the mobile phase is available [18]. The enantioselective analysis of these compounds is not only important from a scientific point of view but can also greatly facilitate the regulatory control of samples. Due to the chiral nature of living organisms, biological systems recognize optical isomers (enantiomers) stereospecifically so that despite their very similar structure, the physiological effect they induce may be different. For both amphetamine and cathinone, it is confirmed that the *S*-enantiomer is more potent than the *R*-enantiomer, but stereospecific effects could also be observed for mephedrone enantiomers [1,19]. However, there are several chiral methods in the literature; a deeper study of their chiral separation mechanism and a comprehensive investigation of the stability of their CD complexes have received less attention so far. Affinity capillary electrophoresis (ACE) is a viable option for analyzing the strength of non-covalent interactions between ligands and analytes [20,21,22,23], besides other methods, e.g., nuclear magnetic resonance (NMR) spectroscopy [24,25]. ACE is a simple, fast, affordable (screening) test to compare the resulting CD–analyte interactions in order to study the chiral separation mechanism and to choose/design an (optimal) antidote candidate. ACE is based on the principle that complexation alters the charge–size ratio of the analyte, which in turn alters the electrophoretic mobility of the analyte.

The ACE method has many benefits, including the ability to conduct preliminary tests quickly, easily, affordably, and with little environmental impact because it only needs a small number of samples and few organic solvents. However, there are limitations; although a large concentration of the analyte aids in detecting the correct peak, it may result in an error in the measurement of *K*. Ligands used for ACE are typically large molecules such as CDs. Adding such molecules to the background electrolyte (BGE) undoubtedly changes the viscosity of the BGE and, thus, the electrophoretic mobility of the ions, thereby biasing the determination of the apparent complex stability constants. Another drawback is that further corrections might also be required in the case of a charged ligand (regarding its ionic strength) to calculate the exact *K* value [21].

The aim of this work is to conduct a comparative analysis of the stability of complexes formed between five selected cathinone analogs and various CD derivatives using affinity capillary electrophoresis. The structure of the formed complexes was planned to be explored by ROESY NMR experiments. In addition to providing systems for chiral separation method development and studying the chiral separation mechanism, our goal is to identify promising CD derivatives with high complex stability. The highly stable complexes between cathinone analogs and CD derivatives may offer opportunities for further studies, potentially leading to the design of targeted antidotes for the relief of cathinone intoxications.

## 2. Results and Discussion

### 2.1. Capillary Electrophoresis

#### 2.1.1. p*K*_a_ Determination of the Cathinone Derivatives by CE-pH Titration

CE is a widely used technique for the analysis of charged compounds; thus, the acid-base properties and the p*K*_a_ of the analyte are crucial in CE method development. Furthermore, the protonation state of compounds has a decisive influence on both their behavior in the body and their affinity for CDs. The advantage of CE-pH titration is that it is fast, simple to perform, does not require any special solvent, and requires only small sample amounts. However, it has the disadvantage that its reproducibility is less than that of conventional pH potentiometry; thus, the results of CE-pH titration should be confirmed by independent method (potentiometry, NMR-pH titration).

The protonation constants of cathinone analogs applying CE-pH titration are summarized in Table 1.

Due to the secondary or tertiary amine moiety, cathinone analogs possess p*K*_a_ in the 8.56–9.00 range (Table 1). Our CE results were confirmed by an independent potentiometric titration, demonstrating consistent results between the two datasets. Notably, Nowak et al. conducted a comprehensive analysis of the acid-base profiles of various cathinone derivatives using CE-pH titration measurements. According to their research, the p*K*_a_ values for mephedrone and MDPV were reported as 8.82 and 9.13, respectively [26]. While our results align with theirs, there is a slight discrepancy of +0.1–0.2 units, possibly attributed to variations in experimental conditions.

Based on the acid-base profiling of the five cathinones, it can be concluded that all cathinone derivatives are positively charged under acidic conditions, and a significant fraction of the molecules are in the cationic (protonated) form under neutral conditions; thus, neutral and charged CDs could also be applied to study their enantioselective complex formation.

#### 2.1.2. Determination of the Cathinone–CD Complex Stabilities by ACE

To assess the CD complex formation capability of the five cathinone derivatives, we devised a screening ACE method tailored to closely mimic physiological conditions. In a preliminary study, the ACE conditions were optimized regarding the type, pH, and concentration of the background electrolyte, the applied voltage, and the injection parameters. The best peak shapes and optimal analysis time could be achieved with a 30 mM phosphate buffer pH 7.4 system (for representative electropherograms, see Figure S1 in the Supplementary Materials). In order to avoid high Joule heat generation, sufficiently low voltage should be used [27]; thus, after registering the Ohm plot (I-U plot), it was set to 15 kV. Throughout our ACE measurements, corrections for viscosity and ionic strength were systematically implemented to ensure precise determination of complex stability constants. Table 2 summarizes the most pertinent calculated apparent and averaged complex stability constants, while additional details on complex mobilities and additional stability values can be found in Table S1 within the Supplementary Materials. In the case of enantioseparation, two complex stability constants are indicated along with the achieved highest enantioresolution (*R_S_*) values and the required selector concentrations.

Comparing the apparent, averaged complex stability constants formed with the three native CDs, the highest stability constants were achieved using the medium cavity-sized β-CD for all five derivatives. A similar cavity-size-dependent tendency could be observed in the case of the carboxymethylated, sulfoalkylated, and sulfated CDs as well (see Table S1 in the Supplementary Materials).

Upon applying neutral beta (or gamma) CD derivatives along with the most commonly used positively charged and *N*-heterocyclic beta CDs [28], no relevant complex formation and enantioseparation improvement could be achieved (see Table S1 in the Supplementary Materials for the results with neutral and positively charged CDs). The most dramatic change in the complex stabilities could be observed by introducing negatively charged substituents; almost all apparent, averaged complex stability constants increased for all five guest molecules. Usually, flephedrone (and the para-substituted analogs) formed fewer stable complexes, while MDPV (and the other methylenedioxy derivative butylone) complexes proved to be the most stable. The effects of the substituent type could be highlighted by the comparison of differently substituted negatively charged CD derivatives. In our ACE measurements, the negatively charged phosphate, carboxylated, and sulfated CD derivatives also formed relatively stable complexes. Comparing the sulfated and sulfoalkylated analogs, the highest complex stabilities could be observed when the sulfate group is directly linked to the CD core. With different alkyl chain lengths, complex stabilities decreased in the order of sulfated > sulfobutylated > sulfopropylated analogs (except for MDPV), and the introduction of a hydroxyl moiety on the sulfopropyl sidechain (SHP analogs) reduced further the complex stability with cathinones. Besides the substituent type, the substitution pattern influenced the complex stabilities, although it is difficult to draw any conclusions from the comparison of randomly substituted cyclodextrins. The favored average degree of substitution was DS~4 in the case of sulfoalkylated CDs; thus, in the complex formation, a four (average) negative charge on the CD molecule seems to be advantageous. The lower DS for SP-β-CD (DS~2) and the higher DS for SBE-β-CD (DS~6.5) were also disadvantageous. However, with the increase in the DS in the case of SBE-β-CD, the enantioselectivity enhanced. The role of the location of the negatively charged substituent on the complexation could be deeply investigated by the set of per-6 single isomer sulfated CD analogs. Compared with randomly substituted S-CDs, these single isomers resulted in lower complex stabilities but with diverse enantiodiscrimination properties (see below). The acetylation of the secondary hydroxyl groups (HDAS) is considered to be unfavorable; furthermore, the sulfation of the primary and the also secondary side (HMDiSu-β-CD) resulted in a significant decrease in the complexation ability of the sulfated CD.

The carboxyalkylation of all cavity-sized CDs resulted in diverse stability values. Comparing CM-β-CD with CE-β-CD, the increase seems to be more significant in the former case with the shorter alkyl chain substituent; however, the substitution pattern of these randomly substituted CDs may also influence the complex stabilities. CM-β-CD reached the highest stability constant with MDPV, followed by butylone. Furthermore, all the carboxymethylated CDs were able to recognize all five cathinone enantiomers and thus may be suitable for chiral separation method development.

Introducing a carboxylate function to the alkyl substituent significantly increased the complex stability of all cathinones: the succinylated derivatives demonstrated outstanding stabilities. Comparing the cathinones based on the complexes formed with Succ-β-CD, the highest stability constants, exceeding 10.000 M^−1,^ were observed for the butylone complex, followed by MDPV, and finally, the para-substituted cathinones. The stability of complexes was influenced by the extent of substitution. Succ-β-CDs with varying degrees of substitution (DS~4 and DS~6) exhibited different stabilities. Lower substitution degrees were found to be favorable for all cathinone derivatives.

Sugammadex (SGX) and its alpha and beta analogs (SAX and SBX) are single isomer per-6-substituted CD derivatives bearing thiopropionic acid substituents at the primary rim. Their complex formation and chiral separation ability were studied earlier in our group [29], and in agreement with our earlier findings, the medium-sized beta analog exhibited the highest affinity for cathinone analogs. Comparing SBX with its *O*-analog, CE-β-CD, remarkable stability enhancement could be achieved with the exchange of the heteroatom and with the per-6-substitution of the primary side instead of the random pattern. Similarly to the Succ-β-CD complexes, outstanding affinity could be observed in the case of the 4-MEC-SBX, butylone-SBX, and MDPV-SBX systems.

Flephedrone displayed the highest complexation constant values with Succ-β-CD (DS~4), but the alpha derivative SAX was found to be an effective complexation agent. Mephedrone reached outstanding stability with SBX, and high stabilities could also be observed with Succ-β-CDs, Phos-β-CD, S-β-CD, and HS-β-CD. 4-MEC demonstrated a similar complexation ability to the previously discussed para-substituted analogs with Succ-β-CD (DS~4) and SBX while also forming a stable complex with SAX (5500 M^−1^). Butylone and MDPV exhibited similar tendencies; they showed the most stable complexes with the succinylated derivatives and SBX (exceeding 5000 M^−1^) but also formed stable complexes with SAX, and several beta derivatives, like S-β-CD, Phos-β-CD, HDMS-β-CD, and the sulfobutylated, sulfopropylated, and carboxymethylated derivatives. Methylenedioxy cathinone derivatives usually showed the highest affinity to the studied CD derivatives; their complex stabilities also exceeded 1000 M^−1^ with the neutral DIME-β-CD.

In light of our results, we can conclude that for all the five studied cathinone analog, the ideal complexing agents (in terms of achieving high stability inclusion complexes) the medium cavity-sized, negatively charged CDs bearing an alkylcarboxylate sidechain. Four out of five studied cathinone analogs exhibited exceptionally high complex stability constants with Succ-β-CD (DS~4); however, most of the cathinones showed also pronounced affinity toward SBX. In the case of mephedrone, SBX proved to be the most suitable derivative.

In several cases, the CD derivatives led to remarkable enantioseparation. The chiral selectivity usually originates from the significant differences between the complex stability constants of the individual enantiomers. For example, pronounced differences could be observed between the complexes of the butylone enantiomers with Succ-β-CD (DS~4). In CE, besides the inclusion of complex stability differences, the complex mobilities may also contribute to the successful enantioseparation. However, the contribution of the complex mobility difference was less significant under the examined conditions (see Table S1 in the Supplementary Materials for the complex mobility values).

#### 2.1.3. Enantioseparation of the Cathinone Derivatives

In CD-based chiral CE separations, the challenge lies in ensuring a matching spatial arrangement of interacting groups, making the success of the separation difficult to predict. While enantioselective complex formation was observed in our ACE measurements, the peak shapes and analysis times were suboptimal for the chiral separation. To offer a suitable CE method for the enantioseparation of all five analogs, further CE experiments were performed.

**Figure 2:**
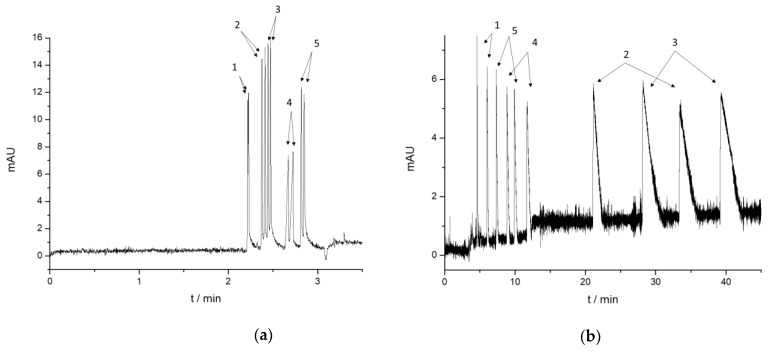

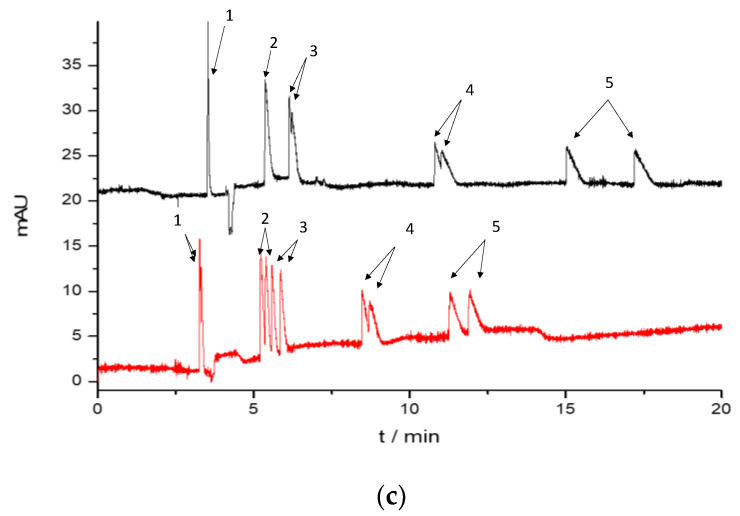
Representative electropherograms applying (**a**) 5 mM CM-γ-CD, (**b**) 5 mM HDAS-β-CD, and (**c**) 5 mM 6-(SB)_7_-β-CD (up) and 5 mM random SBE-β-CD (down) at 20 mM acetate buffer pH 4.5, 25 kV, 215 nm, 25 °C. Analytes: 1 flephedrone, 2 mephedrone, 3 4-MEC, 4 butylone, and 5 MDPV. Further conditions and CD abbreviations can be found in the Section 3.1 and Section 3.2.

In the case of the promising CD systems, CE conditions were optimized regarding the type (phosphate–NaOH, acetate–HCl, acetate–TRIS), pH (2.5–7.0), and concentration (20–100 mM) of the background electrolyte, the injection parameters, and the selector concentration (0.5–10 mM). In the literature, two main BGEs are used for the CD-based chiral analysis of diverse sets of cathinone analogs: the 10–100 mM phosphate buffer pH 2.5(–3.0) [4,7,9,11,12,13,14,16,17,30], and the pH 4.5 acetate buffer [5,6,8]. In our measurements, the best peak shapes and optimal analysis time were provided by 20 mM acetate buffer pH 4.5. The observed enantioseparations are summarized in Table 3. Representative electropherograms are shown in Figure 2 with 5 mM CM-γ-CD, 5 mM HS-β-CD, 4 mM 6-(SB)_7_-β-CD and 4 mM random SBE-β-CD.

Upon comparing the resolutions observed with the two different buffers, no relevant enantioseparation improvement could be achieved with native and neutral CD derivatives applying the pH 4.5 buffer, and the carboxyalkylated and sulfobutylated CDs also resulted in similar *Rs* values; however, these exhibited better peak shapes and shorter analysis time. A fast enantioseparation of all five cathinone derivatives could be achieved in only 3 minutes by applying 5 mM CM-γ-CD. In this system, all the cathinone complexes migrated before the EOF (see Figure 2a). Although CM-γ-CD appeared in method optimization in a previous study [30], its chiral performance and potential as a selector for the separation of cathinone analogs have not been explored. Only its beta analog, the CM-β-CD, has been used for chiral separations of cathinones in the literature [12,14,30].

Upon taking a closer look at the migration order of the five cathinones, usually, the para-substituted cathinone derivatives migrated before the methylenedioxycathinone, among which the fluorinated flephedrone appeared first. The methylenedioxy analogs, particularly MDPV, exhibited delayed migration in the electropherograms due to the notably stable complex formation. This typical migration order, along with the complex formation (and electrodispersion) induced peak distortions, is shown in Figure 2c in the case of random SBE-β-CD and in the case of its single isomer analog, 6-(SB)_7_-β-CD.

The random sulfopropylated and sulfated analogs showed additional selectivities at pH 4.5, although in many cases only with partial separations. The applicability of randomly sulfated and sulfoalkylated CDs for the chiral separation of several cathinone analogs has been demonstrated previously in the literature [5,6,8,9,15]. However, the most dramatic improvements in the chiral selectivities could be observed in the case of single isomer sulfated CDs: HDMS-β-CD was not able to discriminate any of the five cathinone enantiomers at pH 7.4, while at pH 4.5, all of them could be baseline separated with *Rs* values higher than 2. Significant enantioresolution improvement could also be observed in the case of HS-β-CD, HDAS-β-CD, and ODMS-γ-CD, while HMDiSu-β-CD remained ineffective. Unfortunately, even though these CDs could separate all five individual analytes’ enantiomers, overlapping peaks appeared in several cases in their mixture under the examined conditions, e.g., HDMS-β-CD and ODMS-γ-CD systems.

The typical migration order of the cathinone analogs (flephedrone, mephedrone, 4-MEC, butylone, and MDPV) could also be observed in the case of HDMS-β-CD, similarly to randomly substituted S-β-CD. However, it was altered in the case of HS-β-CD (Figure 2b) and HDAS-β-CD; after flephedrone, the methylenedioxy-analogs appeared, and finally, the para-methylated mephedrone and 4-MEC reached the detector. All these single-isomer sulfated CDs possess negatively charged functional groups in the primary side 6-*O* position, and they only differ in their secondary side; the HS-β-CD possess hydroxyl groups, while in the HDAS-β-CD, these OH groups are substituted by acetyl functions, and in the HDMS-β-CD by more hydrophobic methyl groups. These observations demonstrate that subtle changes in the structure of the selector result in alteration in the migration order of the compounds, which may originate from different complex stabilities and/or complex mobilities. Thus, apparent, averaged complex stability constants and complex mobilities were determined under these pH 4.5 conditions also (see Table S3 in Supplementary Materials).

### 2.2. Structural Studies of the Complexes by NMR

**Figure 3:**
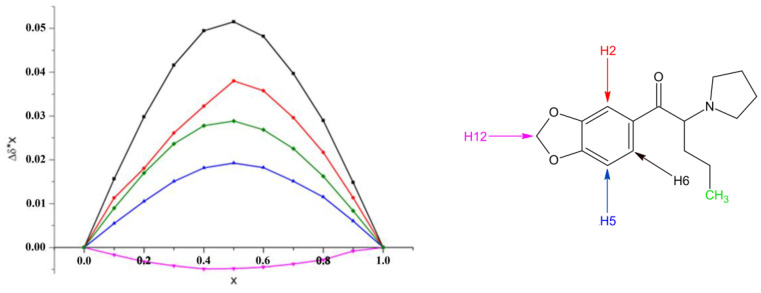
The Job’s plot in the case of MDPV—SBE-β-CD. Further conditions can be found in the Section 3.3. The chemical shift changes in the following protons of MDPV are depicted: black H6, red H2, blue H5, green CH_3_, and rose H12.

The CEval software enables the complex stability constant determination only in the case of complexes with a 1:1 stoichiometry [27]. In order to confirm the average stoichiometry of the inclusion complexes, ^1^H NMR titrations were performed using the continuous variation method (Job’s plot) [31]. The measurements were carried out in the case of para-substituted and methylenedioxy-derived cathinones with both native β-CD and a randomly substituted derivative, SBE-β-CD. It could be clearly observed that the extremes of the curves are at x = 0.5; thus, a stoichiometry of 1:1 could be determined irrespective of the cathinone enantiomers or the applied CDs (Figure 3).

**Figure 4:**
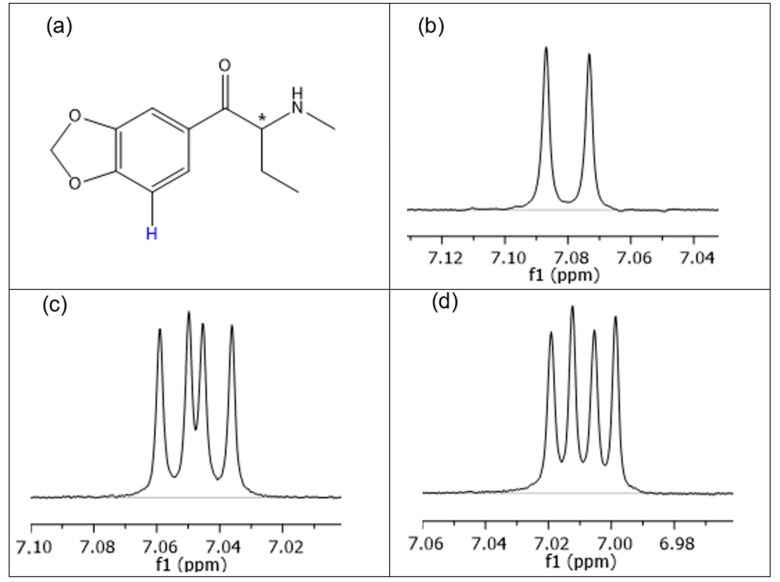
(**a**) The H5 proton of racemic butylone (chiral center denoted by *) and its ^1^H NMR resonance in the presence of (**b**) native β-CD. (**c**) The identical H5 doublet of butylone observed in the presence of SBX, and (**d**) Succ-β-CD displaying two doublets due to diastereomeric splitting (i.e., enantiorecognition) by these two anionic CDs. Further experimental conditions can be found in the Section 3.3.

Chiral recognition has also been observed in the ^1^H NMR spectra of cathinones and selected CDs. Figure 4 illustrates the aromatic (H5) doublet of butylone in the presence of various CDs. In contrast to the native β-CD (see Figure 4b), diastereomeric splitting can be observed in the cases of SBX and Succ-β-CD, indicating the formation of diastereomeric complexes. (see H5 resonance of butylone in Figure 4b–d). Therefore, a single ^1^H NMR experiment is suitable for enantiomeric analysis of cathinones. Unfortunately, in the lack of enantiopure substances, the resonances cannot be assigned to individual enantiomers. Further analysis of enantiomeric recognition of butylone and mephedrone using NMR can be found in the Supplementary Materials (Figures S2–S9).

As CE does not provide any molecular-level information on the interaction between host and guest molecules, NMR measurements were carried out according to previous works [29,32,33,34]. The CD complexation of different cathinone analogs was previously studied by our group in the case of the heptakis(6-*O*-methyl)-β-CD—MDPV system [34] and with the SBX—*N*-ethylbuphedrone [29] complex.

**Figure 5:**
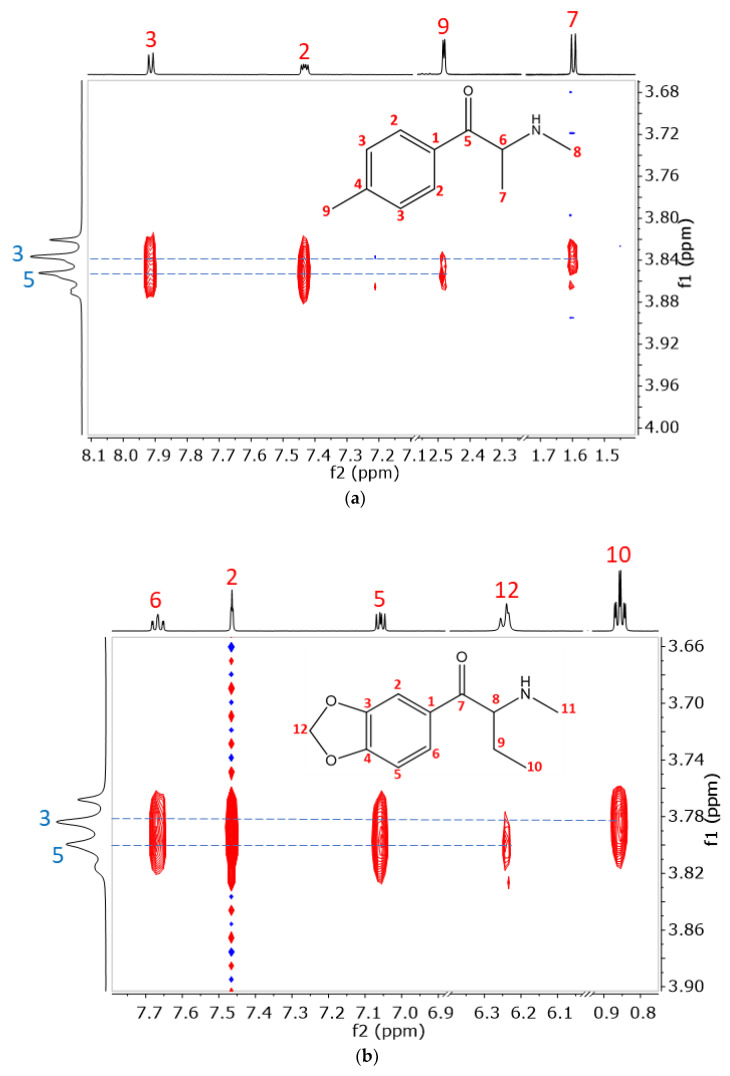
The partial 2D ROESY spectra of (**a**) mephedrone–SBX complex and (**b**) butylone–SBX complex. Further conditions can be found in the Section 3.3.

2D ROESY NMR experiments were carried out to explore the structure of complexes of the cathinone analogs with native β-CD, with Succ-β-CD (possessing outstanding complex stability based on ACE measurements), with the single isomer 6-(SB)_7_-β-CD, and SBX. Intermolecular interactions could be observed between the CD inner cavity protons (H3 and H5) and the aromatic protons of the cathinone derivatives, suggesting the formation of inclusion complexes with SBX (see Figure 5). Similarly, in all other cases, except for the mephedrone–Succ-β-CD system, spatial vicinity could be detected between the cathinones aromatic protons and the CD H3 and H5 cavity protons (see Figures S10–S25 in the Supplementary Materials).

**Figure 6:**
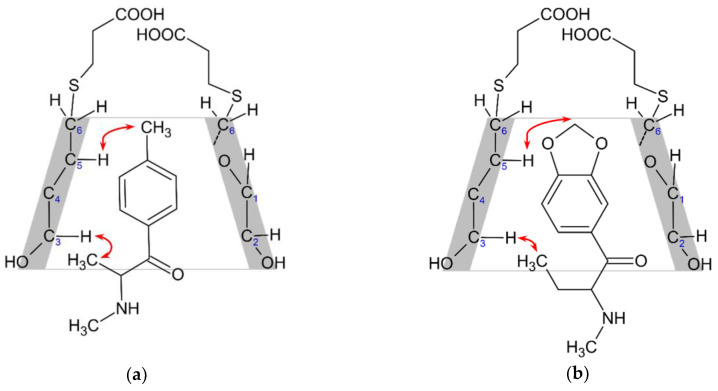
Suggested inclusion complex structure of (**a**) mephedrone–SBX complex and (**b**) butylone–SBX complex based on the ROESY experiments. Red arrows indicate the complex orientation determining spatial proximities; the spatial vicinity between the aromatic protons of the cathinone derivatives and the cyclodextrin cavity protons are not marked.

The orientation of the guest in the CD cavity could also be determined. Figure 5 shows partial 2D ROESY spectra of mephedrone and butylone with SBX. However, all the aromatic ring protons demonstrate spatial vicinity to both cavity protons of the CD with the same intensity, and correlations of the sidechain deliver information about the orientation. In the case of mephedrone (Figure 5a), the methyl group directly attached to the aromatic ring shows correlation only with H5 of SBX, while the methyl group on the alkyl chain is found close to H3, which indicates that the alkyl chain points toward the secondary side of the cone. Similarly, in the case of butylone (Figure 5b), the two methyl groups of the alkyl substituent show a correlation only with H3, indicating that it faces the secondary side of the cavity, while a weak correlation between the methylenedioxy protons and H5 of SBX is also detected, supporting the assigned orientation. For the suggested structure of the inclusion complexes, see Figure 6, where the red arrows indicate the most important spatial proximities determining the orientation of the inclusion complex.

Based on our NMR data (see Figure 5 and Figures S10–S25 in the Supplementary Materials), in all the studied cases, analogous complex structures were revealed. Thus, a similar inclusion-type complex structure can be proposed, as in our previous measurements with the heptakis(6-*O*-methyl)-β-CD–MDPV complex [34].

## 3. Materials and Methods

### 3.1. Materials

The cathinone analogs mephedrone ((±)-2-(methylamino)-1-(4-methylphenyl)propan-1-one), flephedrone (4-FMC, (±)-1-(4-fluorophenyl)-2-(methylamino)propan-1-one) 4-MEC ((±)-2-(ethylamino)-1-(4-methylphenyl)propan-1-one), butylone ((±)-1-(1,3-benzodioxol-5-yl)-2-(methylamino)-butan-1-one), and MDPV (3,4-methylenedioxypyrovalerone, (±)-1-(benzo[d]-[1,3]-dioxol-5-yl)-2-(pyrrolidin-1-yl)pentan-1-one), were purchased from online web shops.

All native CDs (α, β and γ-CD) and their derivatives with various degrees of substitution randomly methylated-α-CD DS~11 (RAME-α-CD), randomly methylated-β-CD DS~12 (RAME-β-CD), randomly methylated-γ-CD DS~12 (RAME-γ-CD), dimethylated-β-CD DS~14 (DIME-β-CD), permethylated-α-CD (TRIME-α-CD), permethylated-β-CD (TRIME-β-CD), permethylated-γ-CD (TRIME-γ-CD), hydroxypropylated-α-CD DS~3 (HP-α-CD), hydroxypropylated-β-CD DS~4.5 (HP-β-CD) and hydroxypropylated-γ-CD DS~3.2 (HP-γ-CD), acetylated-β-CD DS~16 (Ac-β-CD), carboxymethylated-α-CD DS~3.5 (CM-α-CD), carboxymethylated-β-CD DS~3 (CM-β-CD), carboxymethylated-γ-CD DS~4 (CM-γ-CD), carboxyethylated-β-CD DS~3 (CE-β-CD), succinyl-β-CD DS~4 and DS~6 (Succ-β-CD), sualfadex (SAX, hexakis-(6-deoxy-6-(2-carboxyethyl)thio)-α-CD), subetadex (SBX, heptakis-(6-deoxy-6-(2-carboxyethyl)thio)-β-CD), sugammadex (SGX, octakis(6-deoxy-6-(2-carboxyethyl)thio)-γ-CD), phosphated-β-CD DS~2-6 (Phos-β-CD), sulfobutyl-ether-α-CD DS~4 (SBE-α-CD), sulfobutyl-ether-β-CD DS~4, DS~6.5 and DS~10.4 (SBE-β-CD), sulfobutyl-ether-γ-CD DS~4 (SBE-γ-CD), sulfopropylated-β-CD DS~2 and DS~4 (SP-β-CD), sulfopropylated-γ-CD DS~2 (SP-γ-CD), sulfo(2-hydroxy)propylated-β-CD DS~2.5 (SHP-β-CD), sulfo(2-hydroxy)propylated-γ-CD DS~3 (SHP-γ-CD), sulfated-β-CD DS~13 (S-β-CD), sulfated-γ-CD DS~14 (S-γ-CD), heptakis-(6-O-sulfo)-β-CD (HS-β-CD), heptakis-(2,3-O-dimethyl, 6-O-sulfo)-β-CD (HDMS-β-CD), heptakis-(2,3-O-diacethyl, 6-O-sulfo)-β-CD (HDAS-β-CD), heptakis-(2-O-methyl, 3,6-O-disulfo)-β-CD (HMDiSu-β-CD), hexakis-(2,3-O-dimethyl, 6-O-sulfo)-α-CD (HxDMS-α-CD), octakis-(2,3-O-dimethyl, 6-O-sulfo)-γ-CD (ODMS-γ-CD), heptakis-(6-O-sulfobutyl)-β-CD (6-(SB)_7_-β-CD), mono-(6-*N*-amino-6-deoxy)-β-CD (MA-β-CD), mono-6^A^-(3-hydroxy)propylamino-β-CD (HPA-β-CD), 6-monodeoxy-6-pyrrolidine-β-CD (PYR-β-CD), 6-monodeoxy-6-piperidine-β-CD (PIP-β-CD), and mono-6^A^-(*N*-methyl-piperidine)-β-CD (MePIP-β-CD) were products of CycloLab Ltd. (Budapest, Hungary).

D_2_O (99.9% D) and CD_3_COOD (99.5% D) were products of Merck (Darmstadt, Germany) and Cambridge Isotope Laboratories, Inc. (Tewksbury, MA, USA), respectively. Acetic acid, Tris, sodium dihydrogen phosphate (NaH_2_PO_4_), disodium hydrogen phosphate (Na_2_HPO_4_), sodium tetraborate (Na_2_B_4_O_7_), hydrochloric acid (HCl), sodium hydroxide (NaOH), methanol, and dimethyl sulfoxide (DMSO) used for the preparation of buffer solutions, rinsing solutions, or applied as sample solvent or EOF marker were of analytical grade and purchased from commercial suppliers (Sigma-Aldrich, Budapest, Hungary). All reagents were used without further purification. Bidistilled Millipore water was used throughout this study.

### 3.2. Capillary Electrophoresis

The CE experiments were performed on a HP ^3D^CE and on an Agilent 7100 instrument (Agilent Technologies, Waldbronn, Germany) equipped with a photodiode array detector and the Chemstation software for data handling. Untreated fused silica capillaries (50 µm id, 48.5 cm total, 40 cm effective length) were purchased from Agilent. Conditioning of new capillary was conducted by flushing with 1 M NaOH followed by 0.1 M NaOH and water for 30 min each. Prior to all runs, the capillary was preconditioned by rinsing with BGE (2 min). The temperature of the capillary was set to 25 °C. UV detection was performed at 215 nm, and 15–25 kV voltage was applied. Samples were injected hydrodynamically (150 mbar·sec, optimized in the 50–500 mbar·sec range). The CE-pH measurements were carried out applying 25 kV voltage and 200 mbar·sec injection.

The background electrolyte of the CE-pH measurements contained 25 mM Na_2_HPO_4_ and 25 mM Na_2_B_4_O_7_, and the appropriate pH was adjusted with hydrochloric acid. During the ACE measurements, 30 mM phosphate buffer pH 7.4 (30 mM Na_2_HPO_4_—NaOH) was used. The running buffer was 20 mM acetic acid adjusting with 1 M Tris the pH to 4.5 during the chiral screening experiments. The BGEs contained CDs at various concentrations (0.25–20 mM) in the ACE experiments and 1-5-10 mM in the chiral method optimization experiments.

Stock solutions of each cathinone analog (mephedrone, flephedrone, 4-MEC, butylone, and MDPV) were prepared separately in methanol (1 mg/ml), and appropriate dilutions with water were used to prepare sample solutions for the CE-pH titration and for the ACE studies applying DMSO as EOF marker, while their mixture was used during the chiral separation studies.

#### 3.2.1. p*K*_a_ Determination of the Cathinone Derivatives

In order to determine the p*K*_a_ of the five cathinone analogs, CE-pH titration was applied, in which the purpose was to register the effective mobility changes as a function of pH. As the five tested compounds contain secondary or tertiary amino groups, the pH range tested was pH 7–10, applying Na_2_HPO_4_ -Na_2_B_4_O_7_ buffers, where the appropriate pH was adjusted with hydrochloric acid. At a given pH, the mobility is determined by the sum of the mobility of the protonated and deprotonated forms:

(1) $$\mu_{pH}=\mu_{L}x_{L}+\mu_{HL}x_{HL}=\frac{\mu_{L}+\mu_{HL}K\left[H^{+}\right]}{1+K\left[H^{+}\right]}$$

where the *μ_pH_* is the mobility of the compound at the given pH; *μ_L_* and *μ_HL_* are the mobilities of the deprotonated and protonated forms, respectively; *x_L_* and *x_HL_* are the mole fractions of the deprotonated and protonated forms, respectively; and *K* is the protonation constant. Plotting the *μ_pH_* data against pH gives the titration curves from which the protonation constant can be calculated according to Equation (1).

#### 3.2.2. Determination of the Cathinone–CD Complex Stabilities by ACE

The simplest way to determine the mobility is from the migration times (*t_max_*) read from the electropherograms from which the apparent mobility could be calculated [35], but more accurate mobility values could be obtained by the determination with the Haarhoff–van der Linde (HVL) function based on Equation (2).

(2) $$HVL_{\delta }(t;a_{0},a_{1},a_{2},a_{3\delta })=\frac{\frac{a_{0}}{a_{2}a_{3\delta }\sqrt{2\pi }}\exp[\frac{-1}{2}{\left(\frac{t-a_{1}}{a_{2}}\right)}^{2}]}{\frac{1}{\exp(a_{3\delta })-1}+\frac{1}{2}[1+erf(\frac{t-a_{1}}{\sqrt{2}a_{2}})]}$$

where *a_0_* is the area of the HVL function, *a_1_* is the position of the Gaussian component, *a_2_* is the standard deviation of the Gaussian component, and *a_3δ_* is the peak shape distortion. The application of the HVL function is necessary in the case of accurate ACE measurements because, in several cases, the peaks are distorted due to the complex formation, which makes reading the migration times and calculating the mobility more difficult and imprecise [27,36].

The most popular technique for quantifying the analyte–ligand interaction with CE is mobility shift ACE. This technique involves varying the background electrolyte’s ligand concentrations while introducing a constant volume of the analyte and an EOF marker into the capillary. The analyte–ligand equilibrium is assumed to happen before the separation is complete, requiring fast complex formation kinetics. Typical ligand concentrations are 1–2 orders of magnitude greater than the analyte [23]. The following equation describes the observed mobility as a function of the interaction with the following analyte ligand:

(3) $$\mu =\mu_{f}f_{f}+\mu_{c}f_{c}$$

where *μ* is the observed mobility, *μ_f_* and *μ_c_* are the electrophoretic mobility of the free and complexed analyte, and *f_f_* and *f_c_* are the free and ligand-bound fractions of the analyte.

Assuming a 1:1 binding stoichiometry, the effective mobility (*μ_eff_*, calculated by correcting with the EOF marker mobility) can be expressed as

(4) $$\mu_{eff}=\frac{\mu_{f}K+\mu_{c}[L]}{K+[L]}$$

where *K* is the equilibrium dissociation constant and [*L*] is the ligand concentration. The apparent complex stability constant (*K*) can be calculated using several methods, e.g., the X-reciprocal method with linear fitting [37], but non-linear curve fitting based on Equation (4) is more accurate. A hyperbolic growing or decline curve is obtained, and *K* could be determined by plotting the effective mobility as a function of the [*L*] (e.g., CD concentration) in the background electrolyte [38,39]. The CEval software enables faster and simpler evaluation of electropherograms and further data processing to determine the stability constants of complexes with a 1:1 stoichiometric ratio [27].

Increasing the ligand concentration in the BGE alters viscosity and, thus, viscosity correction should be applied to achieve accurate complex stability constants. As the widely used viscosity correction procedure based on the measurement of the current in the CE system is only adequate for neutral ligands, and in our measurements several charged CDs are also studied, an alternative correction method has been chosen [40,41]. The relative viscosity of the CD solutions in phosphate buffer was measured from the time of a 0.1% (*v*/*v*) DMSO sample plug to reach the detection window in a capillary filled with the 30 mM phosphate buffer and the CD containing BGEs by applying a pressure of 100 mbar. The measurements were conducted in triplicate at 200 nm utilizing an Agilent 7100 instrument with untreated fused silica capillaries (50 µm id/48.5 cm/40 cm). Finally, a correction factor was calculated according to Equation (5):

(5) $$v=\frac{\eta^{\left(0\right)}}{\eta }$$

where *η*^(0)^ is the viscosity of the background electrolyte without CD, and *η* is the viscosity of the background electrolyte corresponding to each CD concentration. CEval program offers the possibility of correcting possible viscosity effects with the viscosity slope [27], which is possible according to the following equation:

(6) v=1+k*[L]

where *k* is the viscosity slope.

Besides viscosity correction, the correction of the ionic strength is also required in the case of charged CDs. Constant ionic strength could be achieved with the concentration adjustment of the running buffer: the increase in ionic strength caused by increasing CD concentration was compensated by changing of the phosphate buffer concentration [42].

During the ACE measurement series, the CD concentration is increased whilst injecting a constant amount of the cathinone derivatives and the EOF marker DMSO. The complex stability calculations were conducted with the CEval program applying the HVL function to eliminate the effect of electromigration dispersion on migration time for exact mobility and, thus complex stability constant determination. Viscosity correction and ionic strength correction were applied.

### 3.3. NMR Experiments

The ^1^H NMR spectra were recorded at 298 K on a 600 MHz Varian DDR NMR spectrometer equipped with a 5 mm inverse-detection probe fitted with a gradient module (IDPF probe). The stoichiometry of the complex was determined using 20 mM acetate buffer pH 4.5, prepared with D_2_O. Stock solutions of cathinones, native β-CD, and SBE-β-CD were also prepared at 3 mM, and methanol was added to the solutions as a reference (δ = 3.30 ppm).

Conventional 2D experiments (^1^H-^1^H gCOSY, ROESYAD and ^1^H–^13^C gHSQCAD, and HMBC) for structural elucidation were acquired on 20 mM acetate buffer pH 4.5 solutions containing 1 mM of the cathinone analogs and 2 mM native β-CD, 1 mM Succ-β-CD 1 mM 6-(SB)_7_-β-CD or 1 mM SBX (resulting in a 1:2 or 1:1 cathinone–CD molar ratio). ROESY experiments were recorded with a 300 ms mixing time using a 4.2 kHz spin-lock field. For 1D ROESY selective excitation, the selexcit pulse sequence available in the VnmrJ program was used.

### 3.4. Potentiometry

The protonation constants were confirmed by potentiometry using a GLpKa instrument (Sirius, Forest Row, UK) equipped with a combination Ag/AgCl pH electrode. The four-parameter technique (Four PlusTM method) was used for electrode calibration [43]. The titrations were carried out at constant ionic strength (I = 0.15 M KCl) and temperature (T = 25.0 ± 0.5 °C) under a nitrogen atmosphere. An amount of 10 ml of a ~1 mM aqueous solution of the analytes (4-MEC, MDPV) was pre-acidified to pH 2.0 with 0.5 M HCl and then titrated with 0.5 M KOH to pH 12.2. Three parallel measurements were carried out. The p*K_a_* values were calculated using the RefinementProTM software (Sirius, Forest Row, UK).

## 4. Conclusions

The increase in the number and in the consumption of designer drugs makes an ever-growing challenge for the health sector. While CD derivatives hold promise as ideal candidates for antidotes against various toxic/illicit components due to their complexing properties, selecting the optimal complexing agent for the guest molecules remains a complex task.

In order to characterize the complex formation, a systematic experimental design was developed in which more than 30 neutral and positively and negatively charged CD derivatives were screened and compared according to their cavity size, substituent type and location, and the groups responsible for the favorable interactions were identified and compared in case of the five cathinone derivatives. The succinylated-β-CD and subetadex exhibited outstanding complex stabilities. Our thorough and systematic studies using CE and NMR provided insights into the key factors influencing the complex stability of these components. These measurements may streamline and enhance the predictability of future antidote development studies. The findings of our research thereby hold the potential to pave the way for the development of CD-based antidotes for cathinone analogs in the future.

The enantioselective analysis of cathinone-type compounds holds scientific significance and can also greatly facilitate the development of chiral analytical methods. Following the acid-base profiling of the five cathinones and their CD complexation studies by ACE, herein, we proposed additional promising CD-based systems for the chiral separation of the studied cathinone analogs. Pronounced chiral selectivities were observed with single isomer sulfated CDs, including HDMS-β-CD, HS-β-CD, HDAS-β-CD, and ODMS-γ-CD, which effectively discriminated the cathinone enantiomers at pH 4.5 background electrolyte. Applying CM-γ-CD allowed for a rapid enantioseparation of all five cathinone derivatives in just 3 minutes.

## Figures and Tables

**Table 1:** The determined p*K*_a_ values of the studied cathinone derivatives. Further conditions can be found in Section 3.2 and Section 3.4.

	Mephedrone	Flephedrone	4-MEC	Butylone	MDPV
**CE-pH Titration**	8.61 ± 0.01	8.56 ± 0.01	8.81 ± 0.02	8.64 ± 0.01	9.00 ± 0.02
**Potentiometry**	-	-	8.85 ± 0.04	-	9.05 ± 0.04

**Table 2:** Cathinone–CD complex stability constants (M^−1^) measured by affinity capillary electrophoresis at 30 mM phosphate buffer (pH 7.4), 25 °C, 15 kV, 215 nm. In some cases, CDs with different degrees of substitution (DS) were used for the complexation study. In the case of enantioseparation, the complex stability constants refer to the first (first row) and the second (second row) migrating enantiomer, and the maximal resolution values (*Rs*) are also indicated with the optimal cyclodextrin concentrations. Further conditions and CD abbreviations can be found in the Section 3.1 and Section 3.2. The complex stability constants with the full set of CDs, along with the complex mobilities, are summarized in Table S1 in the Supplementary Materials.

	Cyclodextrin	Flephedrone	Mephedrone	4-MEC	Butylone	MDPV
**Native CDs**	**β-CD**	350 ± 60	560 ± 50	390 ± 45	500 ± 50	1400 ± 135
					430 ± 55 *Rs* 0.7 (7 mM)	810 ± 155 *Rs* 0.8 (8 mM)
**Negative CDs**	**CM-β-CD**	190 ± 10	610 ± 30	620 ± 40	1500 ± 50	1900 ± 90
		225 ± 10 *Rs* 1.3 (10 mM)	615 ± 25 *Rs* 0.7 (3 mM)	725 ± 45 *Rs* 0.6 (10 mM)	1500 ± 60 *Rs* 0.4 (2 mM)	2700 ± 150 *Rs* 3.3 (10 mM)
	**CE-β-CD**	150 ± 10	400 ± 30	590 ± 40	975 ± 95	1560 ± 120
			430 ± 25 *Rs* 0.8 (10 mM)			2000 ± 155 *Rs* 1.6 (10 mM)
	**SAX**	2000 ± 35	530 ± 45	5550 ± 650	2900 ± 200	2550 ± 145 2800 ± 75 *Rs* 1.3 (5 mM)
	**SBX**	575 ± 35 610 ± 45 *Rs* 1.0 (3 mM)	5000 ± 500	8000 ± 340	8250 ± 400 8750 ± 820 *Rs* 0.9 (3 mM)	9650 ± 900 12,830 ± 900 *Rs* 1.6 (3 mM)
	**SGX**	315 ± 20	825 ± 65 1000 ± 100 *Rs* 2.5 (4 mM)	975 ± 90 960 ± 95 *Rs* 1.3 (4 mM)	1700 ± 110	1500 ± 145 2000 ± 185 *Rs* 2.2 (4 mM)
	**Succ-β-CD**	1600 ± 230	1900 ± 200	4500 ± 680	5200 ± 645	5600 ± 175
	**(DS~6)**				6200 ± 870 *Rs* 1.8 (4 mM)	
	**Succ-β-CD**	7200 ± 1000	2750 ± 300	8900 ± 920	13,500 ± 1350	12,100 ± 2150
	**(DS~4)**			8200 ± 880 *Rs* 1.1 (2 mM)	30,500 ± 5300 *Rs* 1.3 (1.5 mM)	10,600 ± 35 *Rs* 2.5 (2 mM)
	**Phos-β-CD**	330 ± 20	1900 ± 200	1200 ± 85	1400 ± 130	870 ± 60
		440 ± 35 *Rs* 4.0 (10 mM)	2100 ± 230 *Rs* 1.8 (10 mM)	1400 ± 120 *Rs* 1.4 (8 mM)	1600 ± 140 *Rs* 1.7 (10 mM)	1200 ± 85 *Rs* 2.6 (3 mM)
	**SBE-β-CD** **(DS~4)**	175 ± 25	300 ± 60	560 ± 60	1600 ± 280	3400 ± 45
			325 ± 60 *Rs* 0.7 (5 mM)	550 ± 40 *Rs* 0.8 (5 mM)		
	**SBE-β-CD** **(DS~6.5)**	200 ± 20	500 ± 20	560 ± 35	1200 ± 60	2300 ± 140
		200 ± 12 *Rs* 1.0 (8 mM)	500 ± 40 *Rs* 0.6 (8 mM)	660 ± 35 *Rs* 1.4 (8 mM)	1300 ± 60 *Rs* 0.7 (8 mM)	2550 ± 200 *Rs* 1.1 (8 mM)
	**SP-β-CD** **(DS~2)**	140 ± 12	390 ± 20	440 ± 25	710 ± 45	1100 ± 60
						1220 ± 50 *Rs* 0.6 (0.8 mM)
	**SP-β-CD** **(DS~4)**	120 ± 12	500 ± 65	620 ± 65	1350 ± 130	3400 ± 60
			450 ± 65 *Rs* 0.5 (7 mM)			
	**S-β-CD**	860 ± 100	2000 ± 110	1450 ± 190	2160 ± 260	2700 ± 250
			2300 ± 85 *Rs* 4.8 (2 mM)	1350 ± 75 *Rs* 5.0 (3 mM)		
	**HS-β-CD**	580 ± 14	1400 ± 175	n.d.	n.d.	n.d.
		470 ± 65 *Rs* 0.7 (3 mM)	1800 ± 25 *Rs* 6.2 (4 mM)			
	**HDAS-β-CD**	110 ± 37	450 ± 60	560 ± 40	725 ± 20	360 ± 55
		360 ± 75 *Rs* 2.8 (5 mM)	730 ± 40 *Rs* 8.6 (5 mM)	830 ± 45 *Rs* 7.9 (5 mM)		340 ± 30 *Rs* 2.3 (5 mM)
	**HDMS-β-CD**	n.d.	n.d.	1670 ± 400	1750 ± 800	915 ± 265

n.d.: not determined.

**Table 3:** Enantioseparation (*Rs*) of cathinones applying various CDs at 20 mM acetate buffer (pH 4.5), 25C, 15 kV, and 215 nm. Further conditions and CD abbreviations can be found in the Section 3.1 and Section 3.2. Enantioseparations with the full set of CDs are summarized in Table S2 in the Supplementary Materials.

	Cyclodextrin	Concentration (mM)	Flephedrone	Mephedrone	4-MEC	Butylone	MDPV
**Negative CDs**	**CM-α-CD**	**1**	0.5	0.6	0.4	0.3	1.7
		**5**	0.9	1.7	1.5	0.5	2.9
		**10**	1.2	0.8	0.6	0.8	5.5
	**CM-β-CD**	**1**	0.4	0	0.3	0.6	1.6
		**5**	0.8	0.1	0.4	0.3	2.1
		**10**	0.8	0.4	0.5	0.4	3.5
	**CM-γ-CD**	**1**	0	1.1	0.7	1.2	0.9
		**5**	0.9	2.6	2.1	1.6	1.9
		**10**	0.9	2.2	1.7	1.9	0.5
	**CE-β-CD**	**1**	0	0.1	0.2	0	0
		**5**	0	0	0	0	1.5
		**10**	0	0.2	0	0	1.1
	**SAX**	**1**	0	0	0	0	0.9
		**5**	0	n.d.	n.d.	0	1.1
		**10**	0	n.d.	n.d.	0	1.8
	**SBX**	**1**	0	0.4	0.5	0	0
		**5**	0.5	0	n.d.	0.7	2.7
		**10**	0.6	0	0.8	0.9	1.9
	**Succ-β-CD** **(DS~4)**	**1** **5** **10**	0 0.9 1.2	0 0 0	0.8 n.d. n.d.	2.4 2.5 2.6	n.d. 0.7 0.7
	**SBE-α-CD**	**1**	0.3	1.5	0.9	0.4	n.d
		**5**	0.4	1.8	1.6	0.9	0.5
		**10**	0.5	2.3	2.2	1.5	0.8
	**SBE-β-CD**	**1**	0.3	0.4	0.5	0.5	0.7
	**(DS~6.5)**	**5**	0.5	0.7	1.2	0.4	1.0
		**10**	0.4	0.8	1.5	0.6	1.4
	**6-(SB)_7_-** **β-CD**	**1** **5** **10**	0 0 0	0 0.2 0.2	0 0.7 0.6	n.d. 0.4 0.6	1.8 2.6 n.d.
	**S-β-CD**	**1**	0.1	1.6	1.9	0.5	0.6
		**5**	0.6	3.1	4.2	n.d.	n.d.
		**10**	0.9	n.d.	n.d.	n.d.	n.d.
	**S-γ-CD**	**1**	0	0.7	0.5	0.6	0
		**5**	0	1.1	0.6	0.7	1.1
		**10**	0	1.0	0	1.4	1.5
	**HS-β-CD**	**1**	3.1	2.9	2.4	3.4	3.4
		**5**	8.1	9.2	8.7	9.2	11.7
		**10**	n.d.	n.d.	n.d.	n.d.	n.d.
	**HDAS-β-CD**	**1**	5.1	2.1	2.7	3.7	1.4
		**5**	13.1	6.2	7.3	9.5	6.1
		**10**	8.0	8.4	7.7	11.4	8.5
	**HDMS-β-CD**	**1**	0.8	0.2	0.3	0.8	0.5
		**5**	1.7	1.6	1.5	2.7	1.9
		**10**	2.6	2.3	2.1	4.0	2.9
	**ODMS-γ-CD**	**1**	0.8	2.2	1.5	1.1	0
		**5**	2.2	5.4	4.0	2.9	1.5
		**10**	3.3	7.5	5.8	4.4	2.7

n.d.: not determined.

## Data Availability

Data are contained within the article and supplementary materials.

## Supplementary Materials

The following supporting information can be downloaded at https://www.mdpi.com/article/10.3390/molecules29040876/s1. Table S1: Cathinone–CD complex stability constants and their mobility values measured by affinity capillary electrophoresis at 30 mM phosphate buffer (pH 7.4); Table S2: Enantioseparation of cathinones applying various CDs; Table S3: Cathinone–CD complex stability constants and complex mobilities measured by affinity capillary electrophoresis at 20 mM acetate buffer (pH 4.5); Figure S1. Representative electropherograms of 4-MEC—S-β-CD complexes in the presence of increasing CD concentration. Further conditions and CD abbreviations can be found in the Section 3.1 and Section 3.2; Figure S2. Selected ^1^H NMR resonances of mephedrone in a 1:1 native β-CD/mephedrone system indicating no diastereotopic splitting (600 MHz, 298 K, D_2_O). Further conditions can be found in the Section 3.3; Figure S3. Selected ^1^H NMR resonances of butylone in a 1:1 native β-CD/butylone system indicating diastereotopic splitting due to the presence of the chiral selector β-CD (600 MHz, 298 K, D_2_O). Further conditions can be found in the Section 3.3; Figure S4. Selected ^1^H NMR resonances of mephedrone in a 2:1 6-(SB)_7_-β-CD/mephedrone system indicating no diastereotopic splitting (600 MHz, 298 K, D_2_O). Further conditions can be found in the Section 3.3; Figure S5. Selected ^1^H NMR resonances of butylone in a 2:1 6-(SB)_7_-β-CD/butylone system indicating diastereotopic splitting due to the presence of the chiral selector 6-(SB)_7_-β-CD (600 MHz, 298 K, D_2_O). Further conditions can be found in the Section 3.3; Figure S6. Selected ^1^H NMR resonances of mephedrone in a 2:1 Succ-β-CD/mephedrone system indicating diastereotopic splitting due to the presence of the chiral selector Succ-β-CD (600 MHz, 298 K, D_2_O). Further conditions can be found in the Section 3.3; Figure S7. Selected ^1^H NMR resonances of butylone in a 2:1 Succ-β-CD/butylone system indicating diastereotopic splitting due to the presence of the chiral selector Succ-β-CD (600 MHz, 298 K, D_2_O). Further conditions can be found in the Section 3.3; Figure S8. Selected ^1^H NMR resonances of mephedrone in a 2:1 SBX/mephedrone system indicating no diastereotopic splitting (600 MHz, 298 K, D_2_O). Further conditions can be found in the Section 3.3; Figure S9. Selected ^1^H NMR resonances of butylone in a 2:1 SBX/butylone system indicating diastereotopic splitting due to the presence of the chiral selector SBX (600 MHz, 298 K, D_2_O). Further conditions can be found in the Section 3.3; Figure S10. The 2D ROESY spectrum of the butylone–β-CD complex. Further conditions can be found in the Section 3.3; Figure S11. The 2D ROESY spectrum of the mephedrone–β-CD complex. Further conditions can be found in the Section 3.3; Figure S12. The 2D ROESY spectrum of the butylone–Succ-β-CD complex. Further conditions can be found in the Section 3.3; Figure S13. The 2D ROESY spectrum of the mephedrone–Succ-β-CD complex. Further conditions can be found in the Section 3.3; Figure S14. The 2D ROESY spectrum of the butylone–6-(SB)_7_-β-CD complex. Further conditions can be found in the Section 3.3; Figure S15. The 2D ROESY spectrum of the mephedrone–6-(SB)_7_-β-CD complex. Further conditions can be found in the Section 3.3; Figure S16. The 2D ROESY spectrum of butylone–SBX complex. Further conditions can be found in the Section 3.3; Figure S17. The 2D ROESY spectrum of the mephedrone–SBX complex. Further conditions can be found in the Section 3.3; Figure S18. The ^1^H NMR spectrum (a) and partial 2D ROESY spectrum (b) of the butylone–β-CD complex. Further conditions can be found in the Section 3.3 Figure S19. The ^1^H NMR spectrum (a) and partial 2D ROESY spectrum (b) of the mephedrone–β-CD complex. Further conditions can be found in the Section 3.3; Figure S20. The ^1^H NMR spectrum (a) and partial 2D ROESY spectrum (b) of the butylone–Succ-β-CD complex. Further conditions can be found in the Section 3.3; Figure S21. The ^1^H NMR spectrum (a) and ^1^H-^13^C HSQC spectrum (b) of the mephedrone–Succ-β-CD complex. Further conditions can be found in the Section 3.3; Figure S22. The ^1^H NMR spectrum (a) and partial 2D ROESY spectrum (b) of the butylone–6-(SB)_7_-β-CD complex. Further conditions can be found in the Section 3.3; Figure S23. The ^1^H NMR spectrum (a) and partial 2D ROESY spectrum (b) of the mephedrone–6-(SB)_7_-β-CD complex. Further conditions can be found in the Section 3.3; Figure S24. The ^1^H NMR spectrum (a) and partial 2D ROESY spectrum (b) of the butylone–SBX complex. Further conditions can be found in the Section 3.3; Figure S25. The ^1^H NMR spectrum (a) and partial 2D ROESY spectrum (b) of the mephedrone–SBX complex. Further conditions can be found in the Section 3.3.
