# Supplementary material for: Cyclodextrin-Enabled Enantioselective Complexation Study of Cathinone Analogs
Source: Molecules. 2024 Feb 16;29(4):876. doi: 10.3390/molecules29040876 (PMC10893103; doi:10.3390/molecules29040876)
Supplement: Supplementary file 1 [file molecules-29-00876-s001.zip › molecules-2846339-supplementary.pdf]

Content: Table S1: Cathinone-CD complex stability constants and their mobility values measured by affinity capillary electrophoresis at 30 mM phosphate buffer (pH 7.4)., **Table S2:** Enantioseparation of cathinones applying various CDs., **Table S3:** Cathinone-CD complex stability constants and complex mobilities measured by affinity capillary electrophoresis at 20 mM acetate buffer (pH 4.5)., **Figure S1.** Representative electropherograms of 4-MEC – S-β-CD complexes in the presence of increasing CD concentration. Further conditions and CD abbreviations can be found in *3.2. Capillary electrophoresis* and *3.1. Materials* section.**Figure S2**. Selected ^{1}H NMR resonances of mephedrone in a 1:1 native β-CD:mephedrone system indicating no diastereotopic splitting (600 MHz, 298 K, D_2O) Further conditions can be found in *3.3 NMR experiments* section. **Figure S3.** Selected ^{1}H NMR resonances of butylone in a 1:1 native β-CD:butylone system indicating diastereotopic splitting due to the presence of the chiral selector β-CD (600 MHz, 298 K, D_2O). Further conditions can be found in *3.3 NMR experiments* section. **Figure S4.** Selected ^{1}H NMR resonances of mephedrone in a 2:1 6-$(SB)_7$-β-CD:mephedrone system indicating no diastereotopic splitting (600 MHz, 298 K, D_2O) Further conditions can be found in *3.3 NMR experiments* section. **Figure S5.** Selected ^{1}H NMR resonances of butylone in a 2:1 6-$(SB)_7$-β-CD:butylone system indicating diastereotopic splitting due to the presence of the chiral selector 6-$(SB)_7$-β-CD (600 MHz, 298 K, D_2O). Further conditions can be found in *3.3 NMR experiments* section. **Figure S6.** Selected ^{1}H NMR resonances of mephedrone in a 2:1 Succ-β-CD:mephedrone system indicating diastereotopic splitting due to the presence of the chiral selector Succ-β-CD (600 MHz, 298 K, D_2O). Further conditions can be found in *3.3 NMR experiments* section. **Figure S7.** Selected ^{1}H NMR resonances of butylone in a 2:1 Succ-β-CD:butylone system indicating diastereotopic splitting due to the presence of the chiral selector Succ-β-CD (600 MHz, 298 K, D_2O). Further conditions can be found in *3.3 NMR experiments* section. **Figure S8.** Selected ^{1}H NMR resonances of mephedrone in a 2:1 SBX:mephedrone system indicating no diastereotopic splitting (600 MHz, 298 K, D_2O) Further conditions can be found in *3.3 NMR experiments* section. **Figure S9.** Selected ^{1}H NMR resonances of butylone in a 2:1 SBX:butylone system indicating diastereotopic splitting due to the presence of the chiral selector SBX (600 MHz, 298 K, D_2O). Further conditions can be found in *3.3 NMR experiments* section. **Figure S10.** The 2D ROESY spectrum of butylone - β-CD complex. Further conditions can be found in *3.3. NMR experiments* section., **Figure S11.** The 2D ROESY spectrum of mephedrone - β-CD complex. Further conditions can be found in *3.3. NMR experiments* section., **Figure S12.** The 2D ROESY spectrum of butylone - Succ-β-CD complex. Further conditions can be found in *3.3. NMR experiments* section., **Figure S13.** The 2D ROESY spectrum of mephedrone - Succ-β-CD complex. Further conditions can be found in *3.3. NMR experiments* section., **Figure S14.** The 2D ROESY spectrum of butylone - 6-$(SB)_7$-β-CD complex. Further conditions can be found in *3.3. NMR experiments* section., **Figure S15.** The 2D ROESY spectrum of mephedrone - 6-$(SB)_7$-β-CD complex. Further conditions can be found in *3.3. NMR experiments* section., **Figure S16.** The 2D ROESY spectrum of butylone - SBX complex. Further conditions can be found in *3.3. NMR experiments* section., **Figure S17.** The 2D ROESY spectrum of mephedrone – SBX complex. Further conditions can be found in *3.3. NMR experiments* section., **Figure S18.** The ^{1}H NMR spectrum **(a)** and partial 2D ROESY spectrum **(b)** of butylone - β-CD complex. Further conditions can be found in *3.3. NMR experiments* section., **Figure S19.** The ^{1}H NMR spectrum **(a)** and partial 2D ROESY spectrum **(b)** of mephedrone – β-CD complex. Further conditions can be found in *3.3. NMR experiments* section., **Figure S20.** The ^{1}H NMR spectrum **(a)** and partial 2D ROESY spectrum **(b)** of butylone - Succ-β-CD complex. Further conditions can be found in *3.3. NMR experiments* section., **Figure S21.** The ^{1}H NMR spectrum **(a)** and ^{1}H-^{13}C HSQC spectrum **(b)** of mephedrone - Succ-β-CD complex. Further conditions can be found in *3.3. NMR experiments* section., **Figure S22.** The ^{1}H NMR spectrum **(a)** and partial 2D ROESY spectrum **(b)** of butylone - 6-$(SB)_7$-β-CD complex. Further conditions can be found in *3.3. NMR experiments* section., **Figure S23.** The ^{1}H NMR spectrum **(a)** and partial 2D ROESY spectrum **(b)** of mephedrone - 6-$(SB)_7$-β-CD complex. Further conditions can be found in *3.3. NMR experiments* section., **Figure S24.** The ^{1}H NMR spectrum **(a)** and partial 2D ROESY spectrum **(b)** of butylone - SBX complex. Further conditions can be found in *3.3. NMR experiments* section., **Figure S25.** The ^{1}H NMR spectrum **(a)** and partial 2D ROESY spectrum **(b)** of mephedrone - SBX complex. Further conditions can be found in *3.3. NMR experiments* section.

Table S1. Cathinone-CD complex stability constants (M^{-1}) and their mobility values measured by affinity capillary electrophoresis at 30 mM phosphate buffer (pH 7.4), 25°C, 15 kV, 215 nm. In the case of enantioseparation the complex stability constants refers to the first (first row) and the second (third row) migrating enantiomer, and the maximal resolution values (*Rs*) are also indicated with the optimal cyclodextrin concentrations. Further conditions and CD abbreviations can be found in *3.1. Materials* section.

Cyclodextrin		Flephedrone	Mephedrone	4-MEC	Butylone	MDPV
**α-CD**	K_{stab1}	40 ± 4	30 ± 4	30 ± 5	40 ± 5	40 ± 6
	μ_{AS1}	10.5 ± 0.8	8.4 ± 0.6	7.1 ± 0.7	5.5 ± 0.7	2.7 ± 1.0
	K_{stab2}					50 ± 5
	μ_{AS2}					3.4 ± 0.7
	Rs					0.6 (30 mM)
**β-CD**	K_{stab1}	350 ± 60	560 ± 50	390 ± 45	500 ± 50	1 400 ± 135
	μ_{AS1}	12.5 ± 0.3	7.4 ± 0.2	5.3 ± 0.3	3.5 ± 0.4	4.6 ± 0.1
	K_{stab2}				430 ± 55	810 ± 155
	μ_{AS2}				2.4 ± 0.5	3.6 ± 0.4
	Rs				0.7 (7 mM)	0.8 (8 mM)
**γ-CD**	K_{stab}	125 ± 10	60 ± 10	90 ± 15	30 ± 3	100 ± 10
	μ_{AS}	13.2 ± 0.1	9.9 ± 0.4	9.2 ± 0.4	2.4 ± 0.6	5.1 ± 0.4
**HP-β-CD**	K_{stab}	15 ± 3	60 ± 4	80 ± 5	170 ± 10	255 ± 10
	μ_{AS}	-5.0 ± 3.7	1.0 ± 0.6	2.4 ± 0.4	2.2 ± 0.2	2.5 ± 0.2
**HP-γ-CD**	K_{stab}	220 ± 35	25 ± 3	40 ± 4	40 ± 5	50 ± 10
	μ_{AS}	15.5 ± 0.2	10.5± 0.6	11.1 ± 0.5	8.7 ± 0.8	6.5 ± 0.1
**RAME-β-CD**	K_{stab1}	40 ± 5	100 ± 5	140 ± 7	325 ± 15	585 ± 35
	μ_{AS1}	3.2 ± 1.4	3.0 ± 0.4	3.2 ± 0.3	3.4 ± 0.1	3.8 ± 0.1
	K_{stab2}				350 ± 15	
	μ_{AS2}				3.7 ± 0.1	
	Rs				0.3 (10 mM)	
**DIME-β-CD**	K_{stab1}	60 ± 6	150 ± 15	210 ± 20	660 ± 70	1 820 ± 190
	μ_{AS1}	2.9 ± 0.5	3.0± 0.4	2.9 ± 0.3	3.9 ± 0.2	4.3 ± 0.2
	K_{stab2}			270 ± 25		
	μ_{AS2}			3.2 ± 0.3		
	Rs			0.6 (10 mM)		
**TRIME-β-CD**	K_{stab}	< 10	20 ± 3	60 ± 15	20 ± 2	40 ± 10
	μ_{AS}	n.d.	8.1 ± 1.4	11.1 ± 0.1	2.5 ± 1.4	9.4 ± 1.4
**TRIME-γ-CD**	K_{stab}	< 10	< 10	20 ± 4	25 ± 10	45 ± 14
	μ_{AS}	n.d.	n.d.	16.1 ± 0.2	15.6 ± 0.2	13.6 ± 0.6
**Ac-β-CD**	K_{stab1}	100 ± 14	220 ± 20	150 ± 15	290 ± 30	310 ± 30
	μ_{AS1}	6.8 ± 1.0	8.5 ± 0.3	6.2 ± 0.5	5.8 ± 0.3	7.4 ± 0.3
	K_{stab2}				380 ± 30	240 ± 20
	μ_{AS2}				5.6 ± 0.2	6.0 ± 0.3
	Rs				0.9 (10 mM)	0.8 (10 mM)
**CM-α-CD**	K_{stab1}	75 ± 9	90 ± 20	150 ± 15	110 ± 12	240 ± 20
	μ_{AS1}	-17.4 ± 2.7	-23.5 ± 4.5	-13.9 ± 1.6	-19.7 ± 2.7	-21.4 ± 0.9
	K_{stab2}	90 ± 15	110 ± 20	150 ± 15	90 ± 12	340 ± 20
	μ_{AS2}	-15.9 ± 2.8	-21.7 ± 3.4	-15.6 ± 1.3	-28.3 ± 4.4	-21.9 ± 0.6
	Rs	0.9 (5 mM)	1.7 (5 mM)	1.4 (5 mM)	0.6 (5 mM)	3.3 (5 mM)

**CM-β-CD**	K_{stab1}	190 ± 10	610 ± 30	620 ± 40	1 500 ± 50	1 900 ± 90
	μ_{AS1}	-17.7 ± 0.8	-18.5 ± 0.4	-18.5 ± 0.5	-18.7 ± 0.2	-18.5 ± 0.3
	K_{stab2}	225 ± 10	615 ± 25	725 ± 45	1 500 ± 60	2 700 ± 150
	μ_{AS2}	-16.7 ± 0.6	-18.5 ± 0.6	-18.2 ± 0.5	-18.9 ± 0.3	-18.8 ± 0.3
	Rs	1.3 (10 mM)	0.7 (3 mM)	0.6 (10 mM)	0.4 (2 mM)	3.3 (10 mM)
**CM-γ-CD**	K_{stab1}	50 ± 9	50 ± 8	65 ± 8	140 ± 10	160 ± 15
	μ_{AS1}	2.5 ± 1.6	-11.8 ± 3.5	-8.1 ± 1.9	-7.6 ± 0.8	-9.0 ± 1.0
	K_{stab2}	30 ± 5	60 ± 10	70 ± 10	170 ± 10	170 ± 17
	μ_{AS2}	-9.4 ± 4.6	-10.5 ± 3.0	-8.4 ± 2.0	-6.9 ± 0.5	-9.0 ± 1.0
	Rs	0.7 (8 mM)	2.6 (10 mM)	1.7 (10 mM)	1.8 (8 mM)	1.4 (7 mM)
**CE-β-CD**	K_{stab1}	150 ± 10	400 ± 30	590 ± 40	975 ± 95	1 560 ± 120
	μ_{AS1}	-8.2 ± 0.7	-13.5 ± 0.6	-12.2 ± 0.4	-14.4 ± 0.5	-14.0 ± 0.3
	K_{stab2}		430 ± 25			2 000 ± 155
	μ_{AS2}		-12.9 ± 0.4			-14.1 ± 0.3
	Rs		0.8 (10 mM)			1.6 (10 mM)
**SAX**	K_{stab1}	2 000 ± 35	530 ± 45	5 550 ± 650	2 900 ± 200	2 550 ± 145
	μ_{AS1}	-13.3 ± 0.2	-16.0 ± 1.0	-5.1 ± 0.4	-9.9 ± 0.5	-18.0 ± 0.3
	K_{stab2}					2 800 ± 75
	μ_{AS2}					-20.1 ± 0.6
	Rs					1.3 (5 mM)
**SBX**	K_{stab1}	575 ± 35	5 000 ± 500	8 000 ± 340	8 250 ± 400	9 650 ± 900
	μ_{AS1}	-34.9 ± 1.2	-32.9 ± 0.9	-32.5 ± 0.4	-31.6 ± 0.4	-32.4 ± 0.7
	K_{stab2}	610 ± 45			8 750 ± 820	12 830 ± 900
	μ_{AS2}	-35.0 ± 1.5			-31.8 ± 0.7	-31.4 ± 0.4
	Rs	1.0 (3 mM)			0.9 (3 mM)	1.6 (3 mM)
**SGX**	K_{stab1}	315 ± 20	825 ± 65	975 ± 90	1 700 ± 110	1 500 ± 145
	μ_{AS1}	-18.7 ± 1.1	-32.1 ± 1.4	-30.7 ± 1.6	-32.3 ± 1.0	-33.8 ± 1.3
	K_{stab2}		1 000 ± 100	960 ± 95		2 000 ± 185
	μ_{AS2}		-30.6 ± 1.5	-31.4 ± 1.7		-33.8 ± 1.2
	Rs		2.5 (4 mM)	1.3 (4 mM)		2.2 (4 mM)
**Succ-β-CD**	K_{stab1}	1 600 ± 230	1 900 ± 200	4 500 ± 680	5 200 ± 645	5 600 ± 175
**(DS~6)**	μ_{AS1}	9.2 ± 0.7	-13.2 ± 1.6	-3.9 ± 1.2	-15.1 ± 1.3	-16.4 ± 0.4
	K_{stab2}				6 200 ± 870	
	μ_{AS2}				-15.0 ± 1.4	
	Rs				1.8 (4 mM)	
**Succ-β-CD**	K_{stab1}	7 200 ± 1000	2 750 ± 300	8 900 ± 920	13 500 ± 1350	12 100 ± 2150
**(DS~4)**	μ_{AS1}	-5.7 ± 0.4	-16.2 ± 0.6	-12.7 ± 0.4	-19.0 ± 0.4	-23.2 ± 1.1
	K_{stab2}			8 200 ± 880	30 500 ± 5300	10 600 ± 35
	μ_{AS2}			-13.6 ± 0.5	-19.1 ± 0.4	-24.1 ± 0.1
	Rs			1.1 (2 mM)	1.3 (1.5 mM)	2.5 (2 mM)
**Phos-β-CD**	K_{stab1}	330 ± 20	1 900 ± 200	1 200 ± 85	1 400 ± 130	870 ± 60
	μ_{AS1}	-25.5 ± 0.9	-27.0 ± 0.7	-26.6 ± 0.7	-27.4 ± 1.1	-30.2 ± 1.3
	K_{stab2}	440 ± 35	2 100 ± 230	1 400 ± 120	1 600 ± 140	1 200 ± 85
	μ_{AS2}	-25.8 ± 1.2	-27.0 ± 0.7	-26.6 ± 0.7	-27.3 ± 0.9	-29.3 ± 1.1
	Rs	4.0 (10 mM)	1.8 (10 mM)	1.4 (8 mM)	1.7 (10 mM)	2.6 (3 mM)
**SBE-α-CD**	K_{stab1}	n.d.	430 ± 70	450 ± 45	220 ± 20	580 ± 80
	μ_{AS1}	n.d.	-18.3 ± 2.4	-17.9 ± 1.0	-31.9 ± 3.3	-18.2 ± 1.6
	K_{stab2}	n.d.	565 ± 80	610 ± 55	530 ± 100	550 ± 90
	μ_{AS2}	n.d.	-16.9 ± 1.7	-17.5 ± 0.8	-13.5 ± 3.4	-20.1 ± 2.2
	Rs		2.5 (7 mM)	2.1 (8 mM)	1.1 (1.5 mM)	0.5 (3 mM)

**SBE-β-CD (DS~4)**	K_{stab1}	175 ± 25	300 ± 60	560 ± 60	1 600 ± 280	3 400 ± 45
	μ_{AS1}	-13.6 ± 2.4	-25.3 ± 3.9	-18.0 ± 1.1	-17.5 ± 1.1	-17.5 ± 0.1
	K_{stab2}		325 ± 60	550 ± 40		
	μ_{AS2}		-25.9 ± 3.6	-18.3 ± 0.8		
	Rs		0.7 (5 mM)	0.8 (5 mM)		
**SBE-β-CD (DS~6.5)**	K_{stab1}	200 ± 20	500 ± 20	560 ± 35	1 200 ± 60	2 300 ± 140
	μ_{AS1}	-17.1 ± 1.3	-24.3 ± 0.4	-24.8 ± 0.7	-25.3 ± 0.4	-26.3 ± 0.4
	K_{stab2}	200 ± 12	500 ± 40	660 ± 35	1 300 ± 60	2 550 ± 200
	μ_{AS2}	-18.1 ± 0.8	-24.7 ± 0.8	-24.2 ± 0.5	-25.6 ± 0.4	-27.0 ± 0.5
	Rs	1.0 (8 mM)	0.6 (8 mM)	1.4 (8 mM)	0.7 (8 mM)	1.1 (8 mM)
**SBE-γ-CD**	K_{stab1}	100 ± 5	70 ± 15	< 10	95 ± 15	160 ± 20
	μ_{AS1}	7.2 ± 0.3	-5.4 ± 3.0	n.d.	-10.0 ± 2.3	-11.6 ± 1.7
	K_{stab2}		65 ± 12		100 ± 15	
	μ_{AS2}		-8.7± 3.3		-11.3 ± 2.3	
	Rs		0.5 (7 mM)		0.6 (7 mM)	
**SP-α-CD**	K_{stab1}	< 10	50 ± 12	200 ± 25	180 ± 30	130 ± 35
	μ_{AS1}	n.d.	-27.9 ± 7.8	-4.8 ± 1.3	-3.5 ± 1.7	-13.2 ± 4.5
	K_{stab2}		100 ± 10	125 ± 20	90 ± 15	180 ± 25
	μ_{AS2}		-13.3 ± 1.7	-12.6 ± 2.5	-15.4 ± 3.9	-12.6 ± 2.0
	Rs		0.4 (5 mM)	0.5 (7 mM)	0.6 (5 mM)	1.3 (5 mM)
**SP-β-CD (DS~2)**	K_{stab1}	140 ± 12	390 ± 20	440 ± 25	710 ± 45	1 100 ± 60
	μ_{AS1}	-9.2 ± 0.5	-9.2 ± 0.5	-9.8 ± 0.5	-13.0 ± 0.5	-13.8 ± 0.3
	K_{stab2}					1 220 ± 50
	μ_{AS2}					-13.4 ± 0.2
	Rs					0.6 (0.8 mM)
**SP-β-CD (DS~4)**	K_{stab1}	120 ± 12	500 ± 65	620 ± 65	1 350 ± 130	3 400 ± 60
	μ_{AS1}	-24.6 ± 2.3	-21.6 ± 1.2	-23.2 ± 1.1	-23.8 ± 1.0	-22.4 ± 0.1
	K_{stab2}		450 ± 65			
	μ_{AS2}		-22.7 ± 1.4			
	Rs		0.5 (7 mM)			
**SP-γ-CD**	K_{stab}	< 10	45 ± 4	70 ± 10	70 ± 10	140 ± 20
	μ_{AS}	n.d.	0.7 ± 0.5	2.2 ± 0.5	-1.1 ± 0.9	-0.3 ± 0.5
**SHP-β-CD**	K_{stab1}	60 ± 10	230 ± 20	250 ± 20	620 ± 35	800 ± 65
	μ_{AS1}	-14.8 ± 3.4	-11.4 ± 0.8	-11.2 ± 0.9	-11.6 ± 0.3	-12.0 ± 0.4
	K_{stab2}		300 ± 20	300 ± 25		910 ± 55
	μ_{AS2}		-9.8 ± 0.6	-9.9 ± 0.7		-11.6 ± 0.3
	Rs		0.6 (3 mM)	0.5 (3 mM)		0.7 (7 mM)
**SHP-γ-CD**	K_{stab1}	45 ± 80	15 ± 5	30 ± 7	30 ± 5	80 ± 10
	μ_{AS1}	6.2 ± 1.4	-23.7 ± 11.4	-7.3 ± 4.3	-15.8 ± 3.5	-7.5 ± 1.7
	K_{stab2}		< 10	25 ± 5	70 ± 7	
	μ_{AS2}		n.d.	-14.7 ± 6.6	-5.8 ± 1.4	
	Rs		0.6 (10 mM)	0.8 (10 mM)	0.7 (10 mM)	
**S-β-CD**	K_{stab1}	860 ± 100	2 000 ± 110	1 450 ± 190	2 160 ± 260	2 700 ± 250
	μ_{AS1}	-15.6 ± 1.0	-25.7 ± 0.8	-28.9 ± 1.2	-31.3 ± 1.6	-33.3 ± 1.0
	K_{stab2}		2 300 ± 85	1 350 ± 75		
	μ_{AS2}		-27.2 ± 0.5	-29.2 ± 0.7		
	Rs		4.8 (2 mM)	5.0 (3 mM)		
**S-γ-CD**	K_{stab}	n.d.	160 ± 15	290 ± 30	960 ± 95	250 ± 55
	μ_{AS}	n.d.	-4.9 ± 1.6	0.1 ± 1.1	8.0 ± 0.3	-11.6 ± 4.1

**HS-β-CD**	K_{stab1}	580 ± 14	1 400 ± 175	n.d.	n.d.	n.d.
	μ_{AS1}	-16.0 ± 0.3	-25.6 ± 1.5	n.d.	n.d.	n.d.
	K_{stab2}	470 ± 65	1 800 ± 25			
	μ_{AS2}	-25.5 ± 2.3	-26.7 ± 0.1			
	Rs	0.7 (3 mM)	6.2 (4 mM)			
**HDAS-β-CD**	K_{stab1}	110 ± 37	450 ± 60	560 ± 40	725 ± 20	360 ± 55
	μ_{AS1}	-16.5 ± 6.5	-37.9 ± 3.0	-31.2 ± 1.1	-29.3 ± 0.3	-23.4 ± 2.5
	K_{stab2}	360 ± 75	730 ± 40	830 ± 45		340 ± 30
	μ_{AS2}		-34.7 ± 0.9	-30.9 ± 0.7		-27.0 ± 1.8
	Rs	2.8 (5 mM)	8.6 (5 mM)	7.9 (5 mM)		2.3 (5 mM)
**HxDMS-α-CD**	K_{stab}	105 ± 12	270 ± 110	55 ± 15	550 ± 95	220 ± 30
	μ_{AS}	0.8 ± 1.2	3.7 ± 3.0	-11.4 ± 6.4	6.5 ± 0.8	1.1 ± 1.1
**HDMS-β-CD**	K_{stab}	n.d.	n.d.	1 670 ± 400	1 750 ± 800	915 ± 265
	μ_{AS}	n.d.	n.d.	9.8 ± 0.5	9.4 ± 0.4	6.1 ± 1.0
**ODMS-γ-CD**	K_{stab1}	90 ± 20	200 ± 35	110 ± 15	145 ± 15	105 ± 7
	μ_{AS1}	-0.5 ± 2.5	5.4 ± 1.1	-1.1 ± 1.6	5.5 ± 0.6	-6.5 ± 1.0
	K_{stab2}	70 ± 10	140 ± 20	125 ± 20	165 ± 25	110 ± 45
	μ_{AS2}	-7.7 ± 2.5	2.4 ± 1.2	-2.4 ± 1.9	3.1 ± 1.1	-8.4 ± 6.2
	Rs	1.1 (5 mM)	2.5 (5 mM)	1.7 (5 mM)	1.6 (5 mM)	0.9 (5 mM)
**HMDiSu-β-CD**	K_{stab}	n.d.	n.d.	260 ± 10	265 ± 20	165 ± 25
	μ_{AS}	n.d.	n.d.	-1.1 ± 0.3	-3.2 ± 0.7	-9.7 ± 2.6
**MA-β-CD**	K_{stab1}	425 ± 80	225 ± 35	180 ± 40	140 ± 25	350 ± 60
	μ_{AS1}	15.2 ± 0.2	12.1 ± 0.3	9.1 ± 0.7	5.7 ± 0.8	7.7 ± 0.4
	K_{stab2}				340 ± 50	200 ± 30
	μ_{AS2}				8.0 ± 0.3	6.7 ± 0.5
	Rs				0.5 (5 mM)	0.7 (5 mM)
**HPA-β-CD**	K_{stab1}	500 ± 105	140 ± 35	145 ± 15	85 ± 15	840 ± 65
	μ_{AS1}	16.0 ± 0.1	11.8 ± 0.6	10.3 ± 0.3	3.8 ± 1.3	11.0 ± 0.1
	K_{stab2}					570 ± 80
	μ_{AS2}					9.1 ± 0.2
	Rs					0.6 (8 mM)
**PYR-β-CD**	K_{stab1}	640 ± 145	115 ± 20	215 ± 30	1 280 ± 170	105 ± 15
	μ_{AS1}	15.1 ± 0.1	10.6 ± 0.8	11.3 ± 0.4	10.8 ± 0.2	6.2 ± 0.7
	K_{stab2}					430 ± 50
	μ_{AS2}					9.1 ± 0.3
	Rs					0.5 (5 mM)
**PIP-β-CD**	K_{stab}	< 10	< 10	< 10	< 10	< 10
	μ_{AS}	n.d.	n.d.	n.d.	n.d.	n.d.
**MePIP-β-CD**	K_{stab}	40 ± 3	45 ± 5	50 ± 5	40 ± 3	85 ± 5
	μ_{AS}	8.5 ± 0.2	7.1 ± 0.4	6.2± 0.3	3.1 ± 0.4	3.9 ± 0.3

n.d.: not determined.

Table S2. Enantioseparation (*Rs*) of cathinones applying various CDs at 20 mM acetate buffer (pH 4.5), 25C, 15 kV, 215 nm. Further conditions and CD abbreviations can be found in *3.1. Materials* section.

Cyclodextrin	Concentration (mM)	Flephedrone	Mephedrone	4-MEC	Butylone	MDPV
**α-CD**	**1-5**	0	0	0	0	0
	**10**	0	0	0.3	0	0
**β-CD**	**1**	0	0	0	0	0.3
	**5**	0	0	0	0	0.8
	**10**	0	0	0	0	0.5
**γ-CD**	**1-5**	0	0	0	0	0
	**10**	n.d.	n.d.	n.d.	n.d.	n.d.
**HP-α-CD**	**1-5**	0	0	0	0	0
	**10**	0	0.3	0.6	0	0.3
**HP-β-CD**	**1**	0	0	0	0	0
	**5**	0	0.3	0.3	0	0.5
	**10**	0	0.3	0.3	0	0.4
**HP-γ-CD**	**1-5-10**	0	0	0	0	0
**RAME-α-CD**	**1-5-10**	0	0	0	0	0
**RAME-β-CD**	**1-5-10**	0	0	0	0	0
**RAME-γ-CD**	**1-5-10**	0	0	0	0	0
**DIME-β-CD**	**1**	0	0	0	0	0
	**5**	0.8	0	0	0	0
	**10**	0.5	0	0	0	0
**TRIME-α-CD**	**1-5**	0	0	0	0	0
	**10**	0.6	0.7	0.8	0	0
**TRIME-β-CD**	**1-5-10**	0	0	0	0	0
**TRIME-γ-CD**	**1-5-10**	0	0	0	0	0
**CM-α-CD**	**1**	0.5	0.6	0.4	0.3	1.7
	**5**	0.9	1.7	1.5	0.5	2.9
	**10**	1.2	0.8	0.6	0.8	5.5
**CM-β-CD**	**1**	0.4	0	0.3	0.6	1.6
	**5**	0.8	0.1	0.4	0.3	2.1
	**10**	0.8	0.4	0.5	0.4	3.5
**CM-γ-CD**	**1**	0	1.1	0.7	1.2	0.9
	**5**	0.9	2.6	2.1	1.6	1.9
	**10**	0.9	2.2	1.7	1.9	0.5
**CE-β-CD**	**1**	0	0.1	0.2	0	0
	**5**	0	0	0	0	1.5
	**10**	0	0.2	0	0	1.1
**SAX**	**1**	0	0	0	0	0.9
	**5**	0	n.d.	n.d.	0	1.1
	**10**	0	n.d.	n.d.	0	1.8
**SBX**	**1**	0	0.4	0.5	0	0
	**5**	0.5	0	n.d.	0.7	2.7
	**10**	0.6	0	0.8	0.9	1.9
**SGX**	**1**	0	0	0.4	0	2.4
	**5**	0	0.8	0.9	0	0
	**10**	0.6	0.7	0.9	0	0

**Succ-β-CD**	**1**	0	0	0.8	2.4	n.d.
**(DS~4)**	**5**	0.9	0	n.d.	2.5	0.7
	**10**	1.2	0	n.d.	2.6	0.7
**SBE-α-CD**	**1**	0.3	1.5	0.9	0.4	n.d
	**5**	0.4	1.8	1.6	0.9	0.5
	**10**	0.5	2.3	2.2	1.5	0.8
**SBE-β-CD**	**1**	0.3	0.4	0.5	0.5	0.7
**(DS~6.5)**	**5**	0.5	0.7	1.2	0.4	1.0
	**10**	0.4	0.8	1.5	0.6	1.4
**SBE-γ-CD**	**1**	0	0	0	0	0
	**5**	0	1.1	1.1	0.5	0.6
	**10**	0	0.9	1.1	0.8	0
**6-(SB)$_7$-β-CD**	**1**	0	0	0	n.d.	1.8
	**5**	0	0.2	0.7	0.4	2.6
	**10**	0	0.2	0.6	0.6	n.d.
**SP-β-CD**	**1**	0	0	0	0.5	0.3
**(DS~4)**	**5**	0	0.4	0.3	0.3	0.4
	**10**	0	0.5	0.4	0	0.6
**SP-γ-CD**	**1**	0	0	0	0	0
	**5**	0	0	0	0	0
	**10**	0	0.8	0.9	0.4	0
**S-β-CD**	**1**	0.1	1.6	1.9	0.5	0.6
	**5**	0.6	3.1	4.2	n.d.	n.d.
	**10**	0.9	n.d.	n.d.	n.d.	n.d.
**S-γ-CD**	**1**	0	0.7	0.5	0.6	0
	**5**	0	1.1	0.6	0.7	1.1
	**10**	0	1.0	0	1.4	1.5
**HS-β-CD**	**1**	3.1	2.9	2.4	3.4	3.4
	**5**	8.1	9.2	8.7	9.2	11.7
	**10**	n.d.	n.d.	n.d.	n.d.	n.d.
**HDAS-β-CD**	**1**	5.1	2.1	2.7	3.7	1.4
	**5**	13.1	6.2	7.3	9.5	6.1
	**10**	8.0	8.4	7.7	11.4	8.5
**HDMS-β-CD**	**1**	0.8	0.2	0.3	0.8	0.5
	**5**	1.7	1.6	1.5	2.7	1.9
	**10**	2.6	2.3	2.1	4.0	2.9
**ODMS-γ-CD**	**1**	0.8	2.2	1.5	1.1	0
	**5**	2.2	5.4	4.0	2.9	1.5
	**10**	3.3	7.5	5.8	4.4	2.7
**HMDiSu-β-CD1-5-10**		0	0	0	0	0

n.d.: not determined.

Table S3. Cathinone-CD complex stability constants (M^{-1}) and complex mobilities (10^{-5} cm^{-2} V^{-1} s^{-1})) measured by affinity capillary electrophoresis at 20 mM acetate buffer (pH 4.5), 25C, 15 kV, 215 nm. The complex stability constants refer to the first (K_{stab1}) and the second (K_{stab2}) migrating enantiomer. Further conditions and CD abbreviations can be found in *3.1. Materials* section.

**Cyclodextrin**		**Flephedrone**	**Mephedrone**	**4-MEC**	**Butylone**	**MDPV**
**HS-β-CD**	K_{stab1}	635 ± 35	2 000 ± 145	2 100 ± 80	1 020 ± 90	500 ± 45
	μ_{AS1}	-23.7 ± 0.8	-31.9 ± 1.3	-30.5 ± 0.7	-29.7 ± 1.8	-35.7 ± 2.0
	K_{stab2}	615 ± 30	2 070 ± 130	1 410 ± 80	1 800 ± 90	1 300 ± 40
	μ_{AS2}	-30.4 ± 1.0	-36.8 ± 1.4	-40.9 ± 1.5	-28.1 ± 0.9	-27.8 ± 0.6
**HDAS-β-CD**	K_{stab1}	470 ± 40	2 400 ± 220	1 400 ± 145	1 200 ± 40	800 ± 50
	μ_{AS1}	-11.2 ± 1.2	-12.2 ± 0.6	-28.0 ± 2.3	-26.1 ± 0.5	-24.0 ± 0.9
	K_{stab2}	660 ± 55	1 500 ± 140	2 100 ± 140	2 000 ± 100	960 ± 80
	μ_{AS2}	-19.9 ± 1.3	-17.9 ± 0.8	-30.9 ± 1.3	-22.8 ± 0.5	-24.3 ± 1.1
**HDMS-β-CD**	K_{stab1}	250 ± 7	205 ± 20	170 ± 15	150 ± 10	160 ± 15
	μ_{AS1}	2.6 ± 0.3	-2.0 ± 1.3	-3.0 ± 1.0	-5.9 ± 0.9	-4.8 ± 1.8
	K_{stab2}	235 ± 20	205 ± 15	190 ± 15	180 ± 12	175 ± 12
	μ_{AS2}	-0.4 ± 1.2	-3.0 ± 0.8	-3.0 ± 0.9	-5.9 ± 1.0	-5.7 ± 1.0

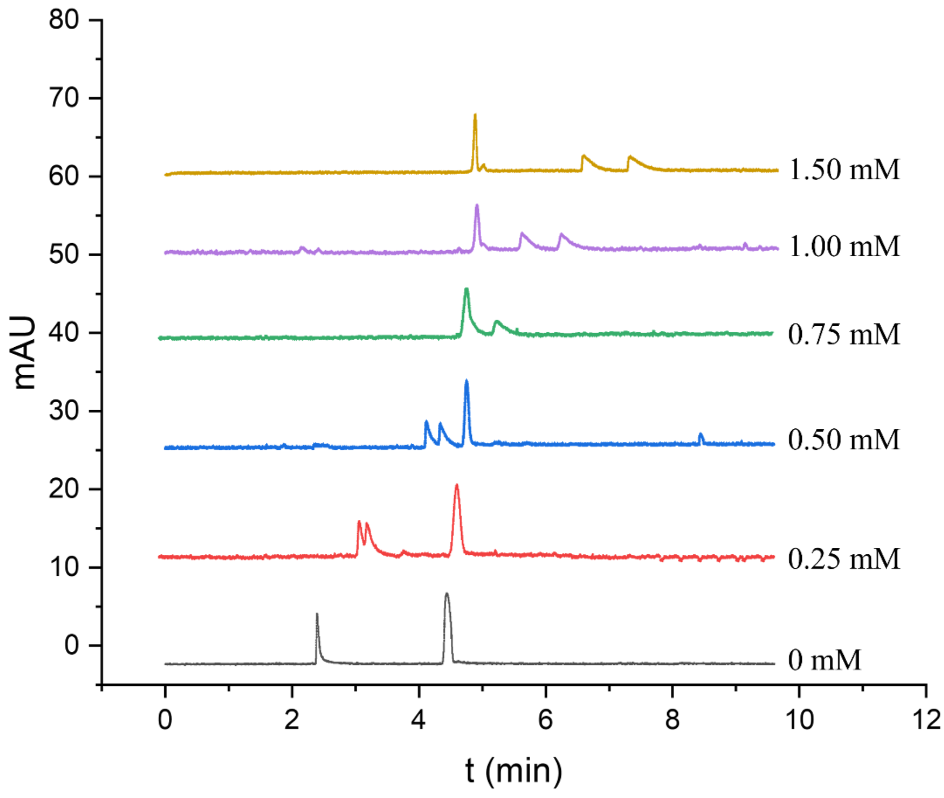

Figure S1. Representative electropherograms of 4-MEC – S-β-CD complexes in the presence of increasing CD concentration. Further conditions and CD abbreviations can be found in *3.2. Capillary electrophoresis* and *3.1. Materials* section.

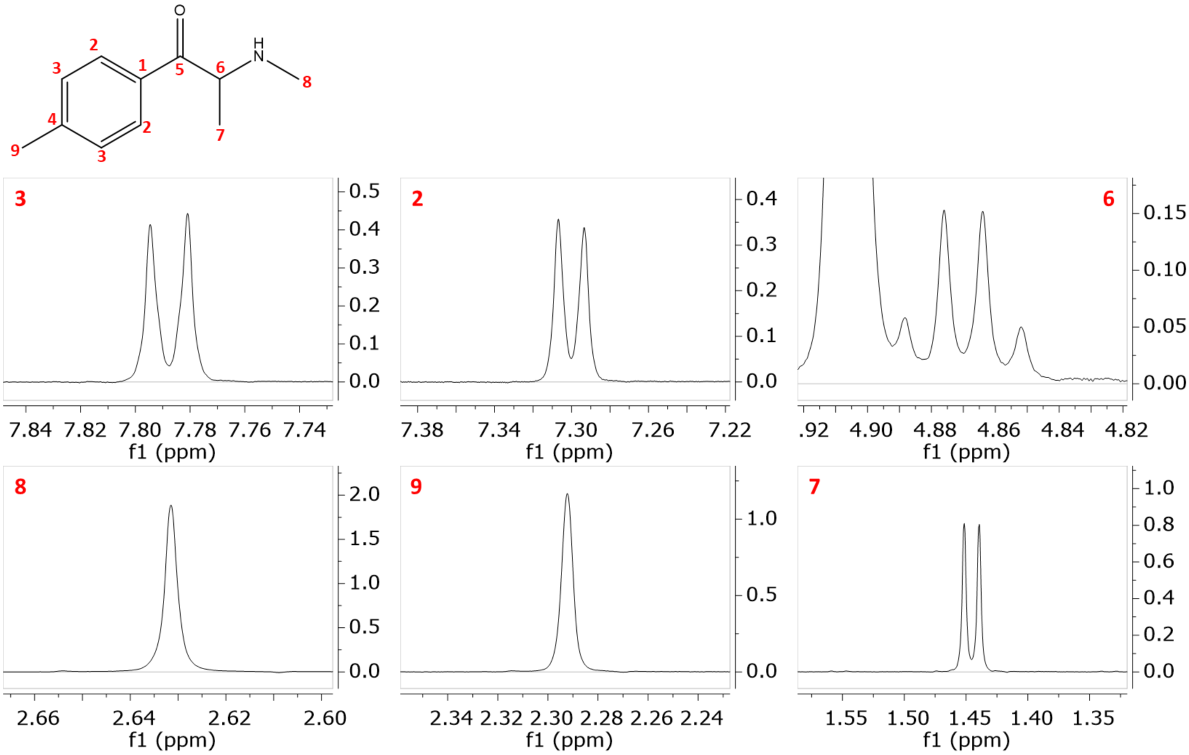

Figure S2. Selected ^{1}H NMR resonances of mephedrone in a 1:1 native β-CD:mephedrone system indicating no diastereotopic splitting (600 MHz, 298 K, D_2O) Further conditions can be found in *3.3 NMR experiments* section.

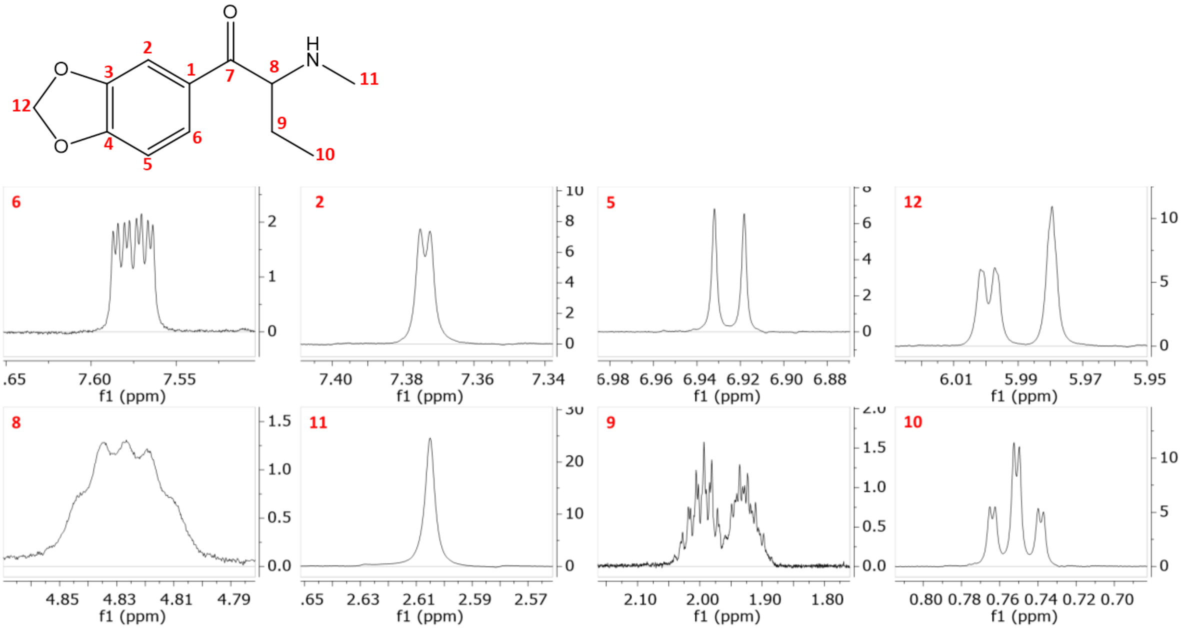

Figure S3. Selected ^{1}H NMR resonances of butylone in a 1:1 native β-CD:butylone system indicating diastereotopic splitting due to the presence of the chiral selector β-CD (600 MHz, 298 K, D_2O). Further conditions can be found in *3.3 NMR experiments* section.

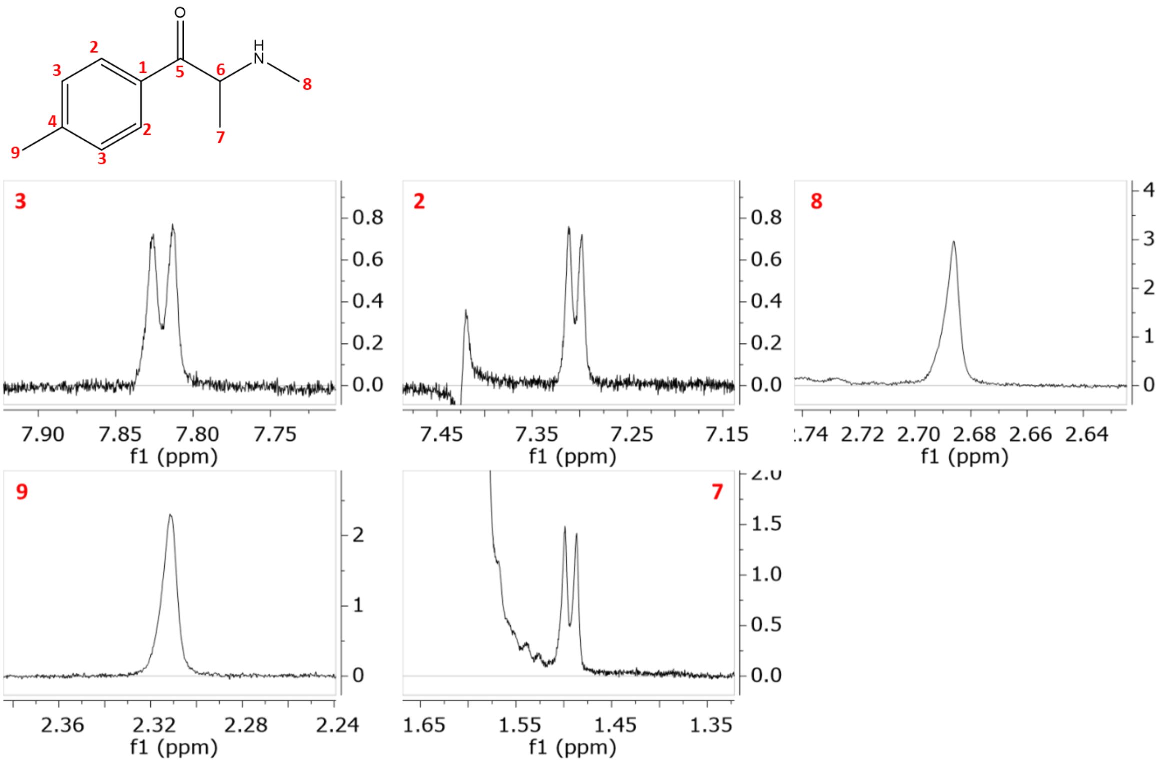

Figure S4. Selected ^{1}H NMR resonances of mephedrone in a 2:1 6-(SB)$_7$-β-CD:mephedrone system indicating no enantiomeric recognition (600 MHz, 298 K, D_2O) Further conditions can be found in *3.3 NMR experiments* section.

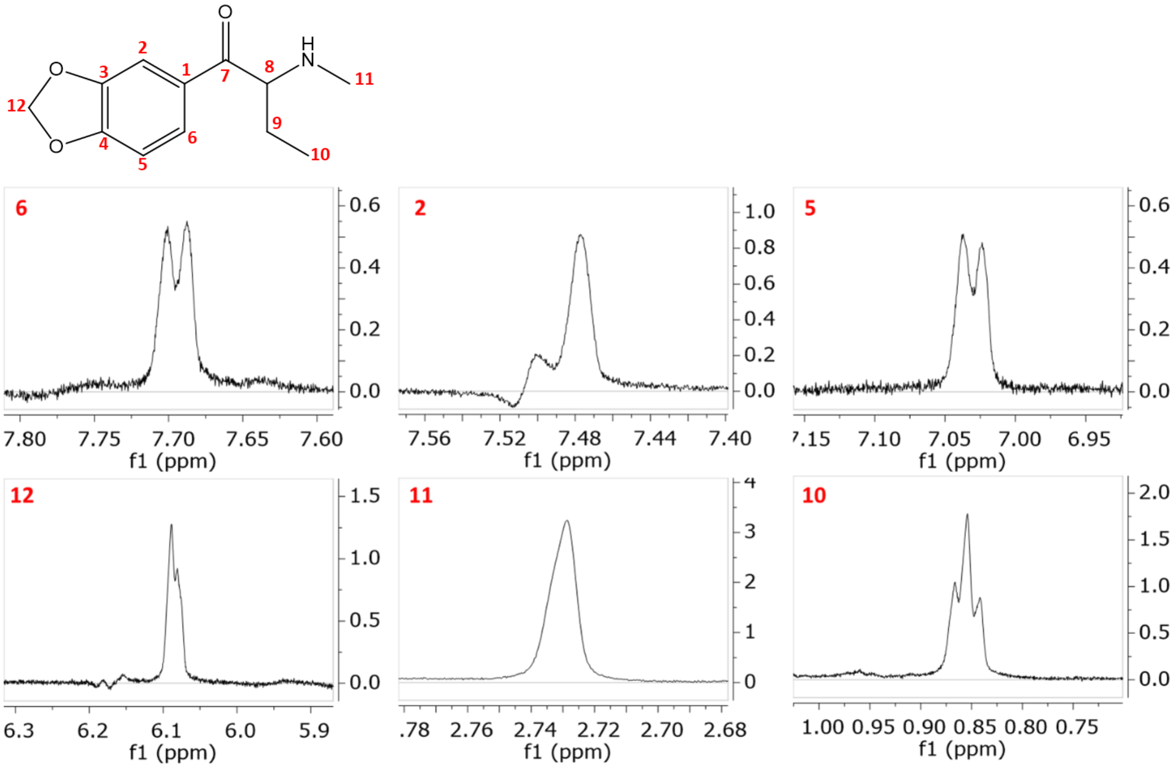

Figure S5. Selected ^{1}H NMR resonances of butylone in a 2:1 6-(SB)$_7$-β-CD:butylone system indicating enantiomeric recognition due to the presence of the chiral selector 6-(SB)$_7$-β-CD (600 MHz, 298 K, D_2O). Further conditions can be found in *3.3 NMR experiments* section.

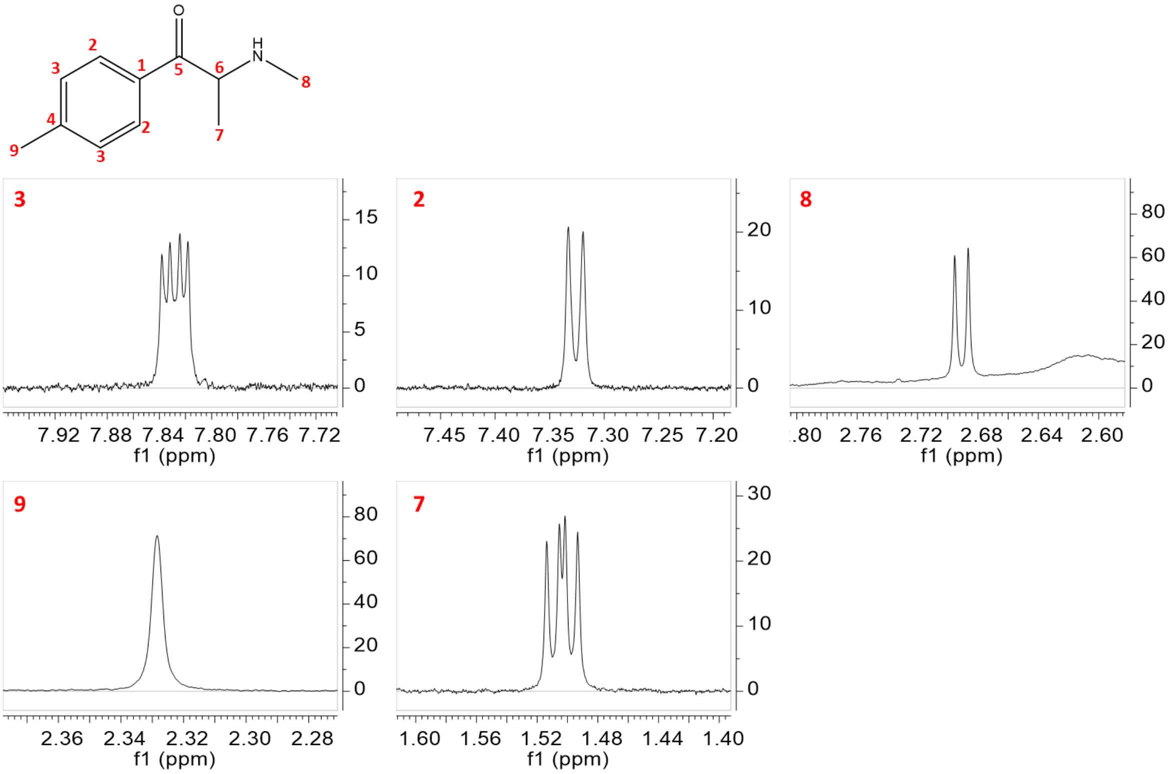

Figure S6. Selected ^{1}H NMR resonances of mephedrone in a 2:1 Succ-β-CD:mephedrone system indicating enantiomeric recognition due to the presence of the chiral selector Succ-β-CD (600 MHz, 298 K, D_2O). Further conditions can be found in *3.3 NMR experiments* section.

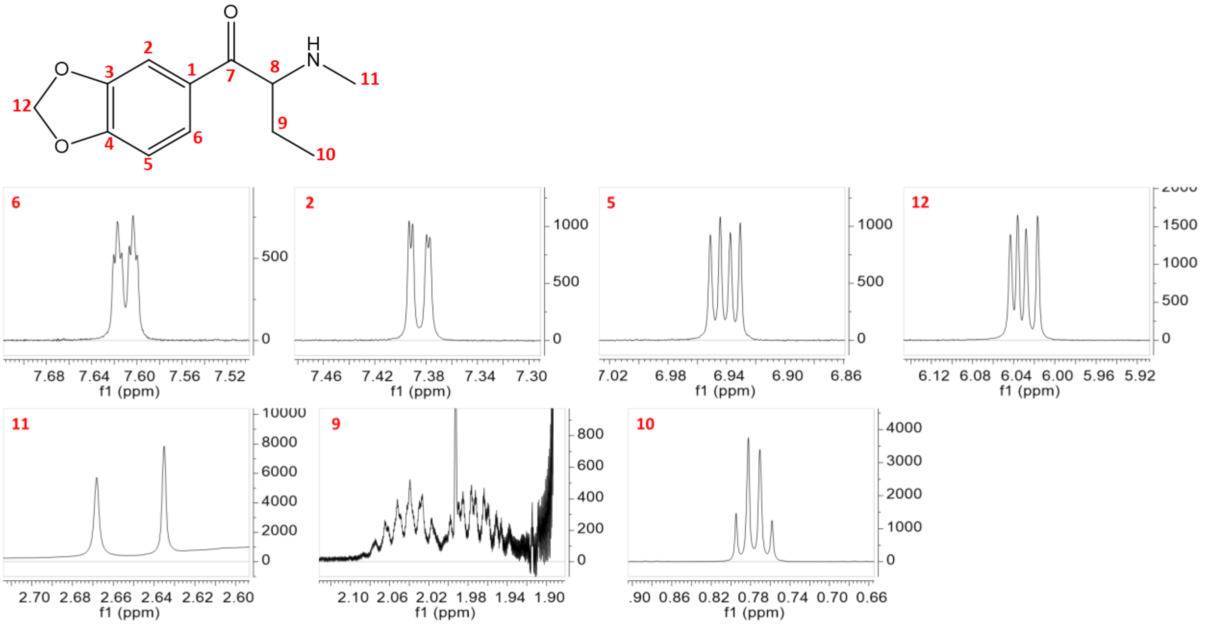

Figure S7. Selected ^{1}H NMR resonances of butylone in a 2:1 Succ-β-CD:butylone system indicating enantiomeric recognition due to the presence of the chiral selector Succ-β-CD (600 MHz, 298 K, D_2O). Further conditions can be found in *3.3 NMR experiments* section.

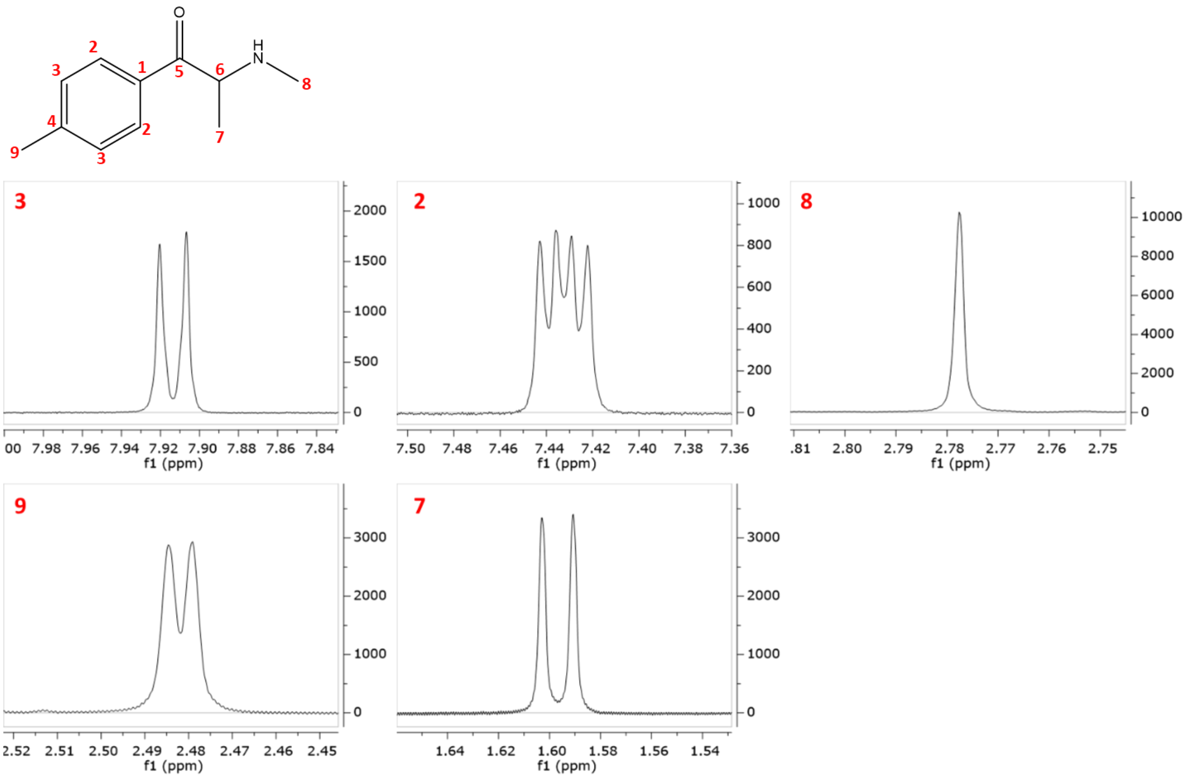

Figure S8. Selected ^{1}H NMR resonances of mephedrone in a 2:1 SBX:mephedrone system indicating no enantiomeric recognition (600 MHz, 298 K, D_2O) Further conditions can be found in *3.3 NMR experiments* section.

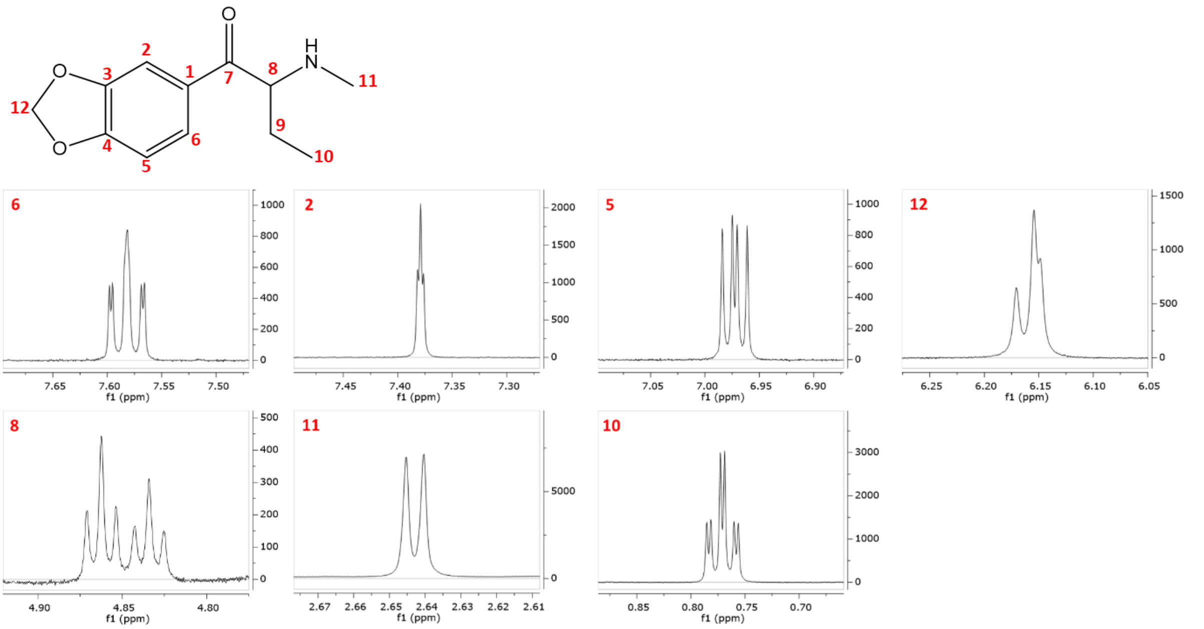

Figure S9. Selected ^{1}H NMR resonances of butylone in a 2:1 SBX:butylone system indicating enantiomeric recognition due to the presence of the chiral selector SBX (600 MHz, 298 K, D_2O). As presaturation was applied to diminish the water resonance, the nearby signals exhibit distortion integrals (see e.g. H8). Further conditions can be found in *3.3 NMR experiments* section.

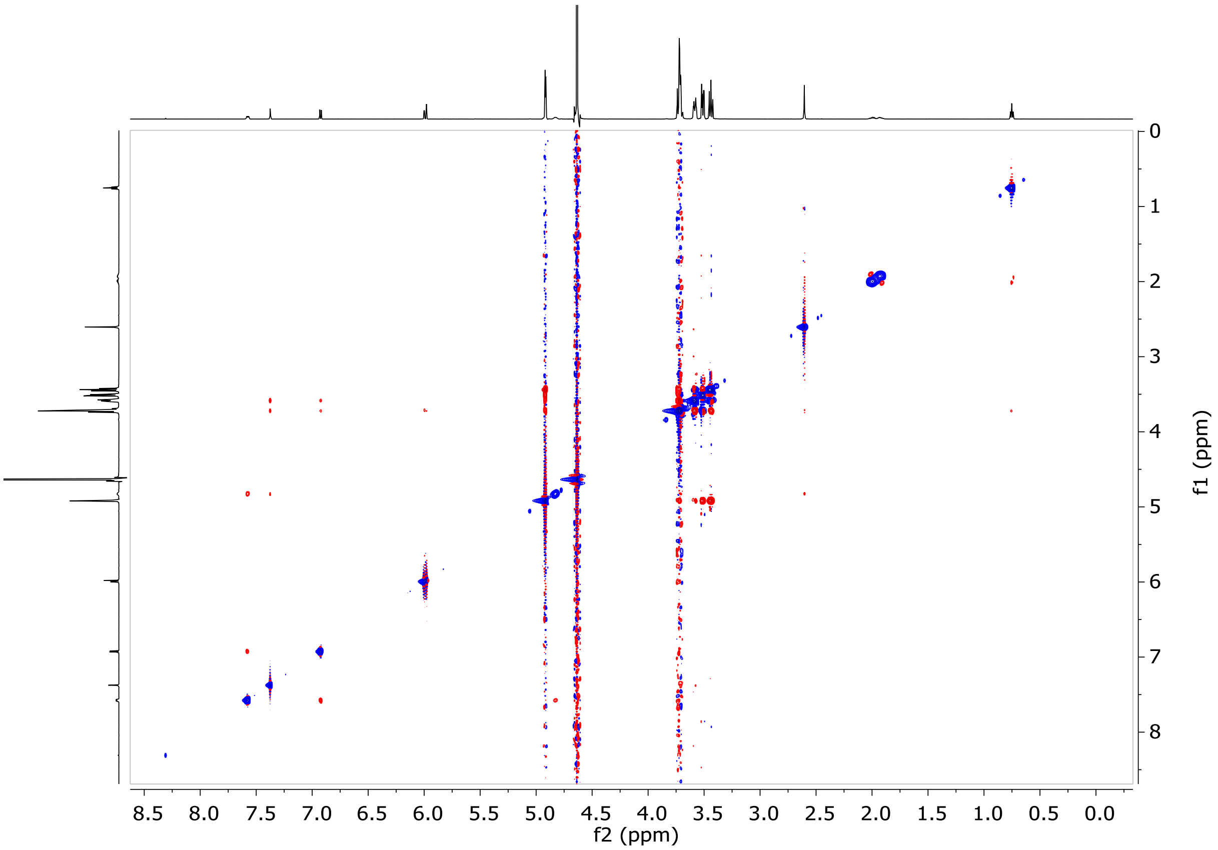

Figure S10. The 2D ROESY spectrum of butylone - β-CD complex. Further conditions can be found in *3.3. NMR experiments* section.

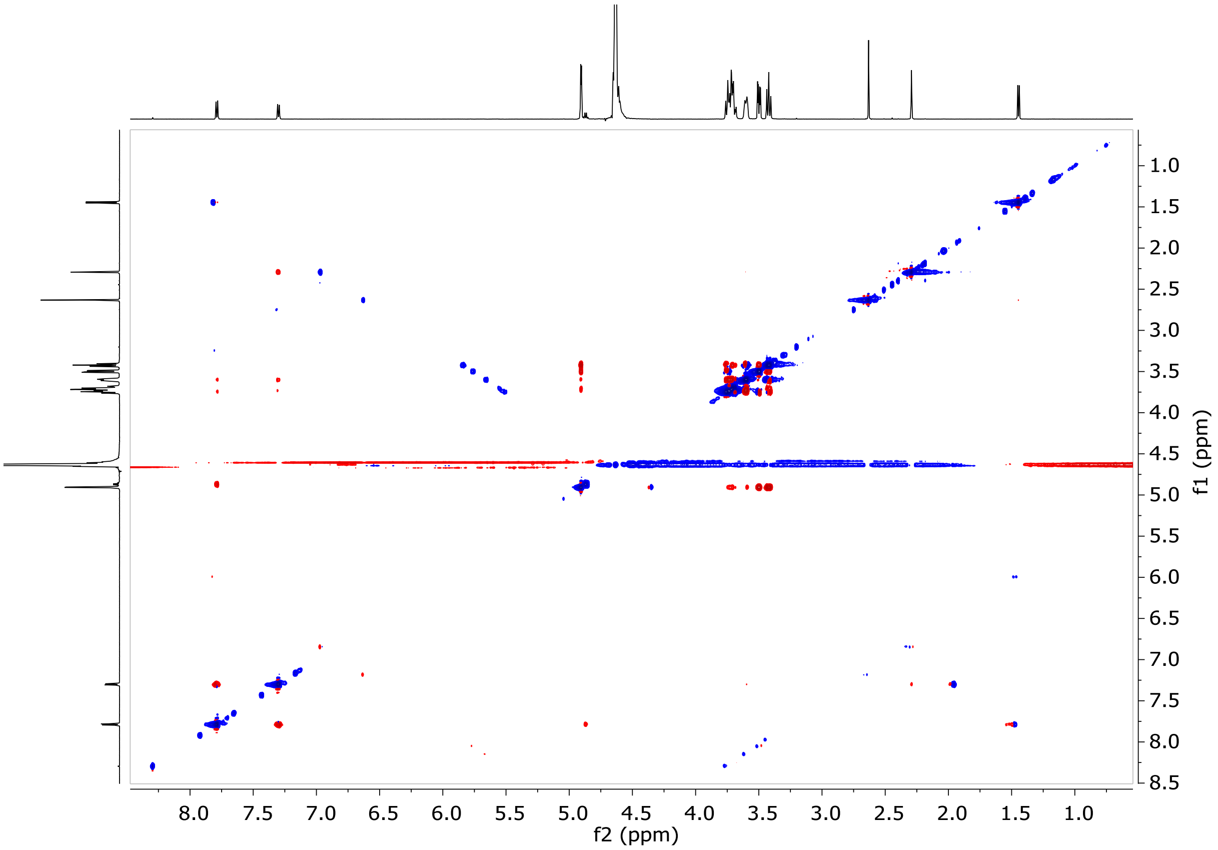

Figure S11. The 2D ROESY spectrum of mephedrone - β-CD complex. Further conditions can be found in *3.3. NMR experiments* section.

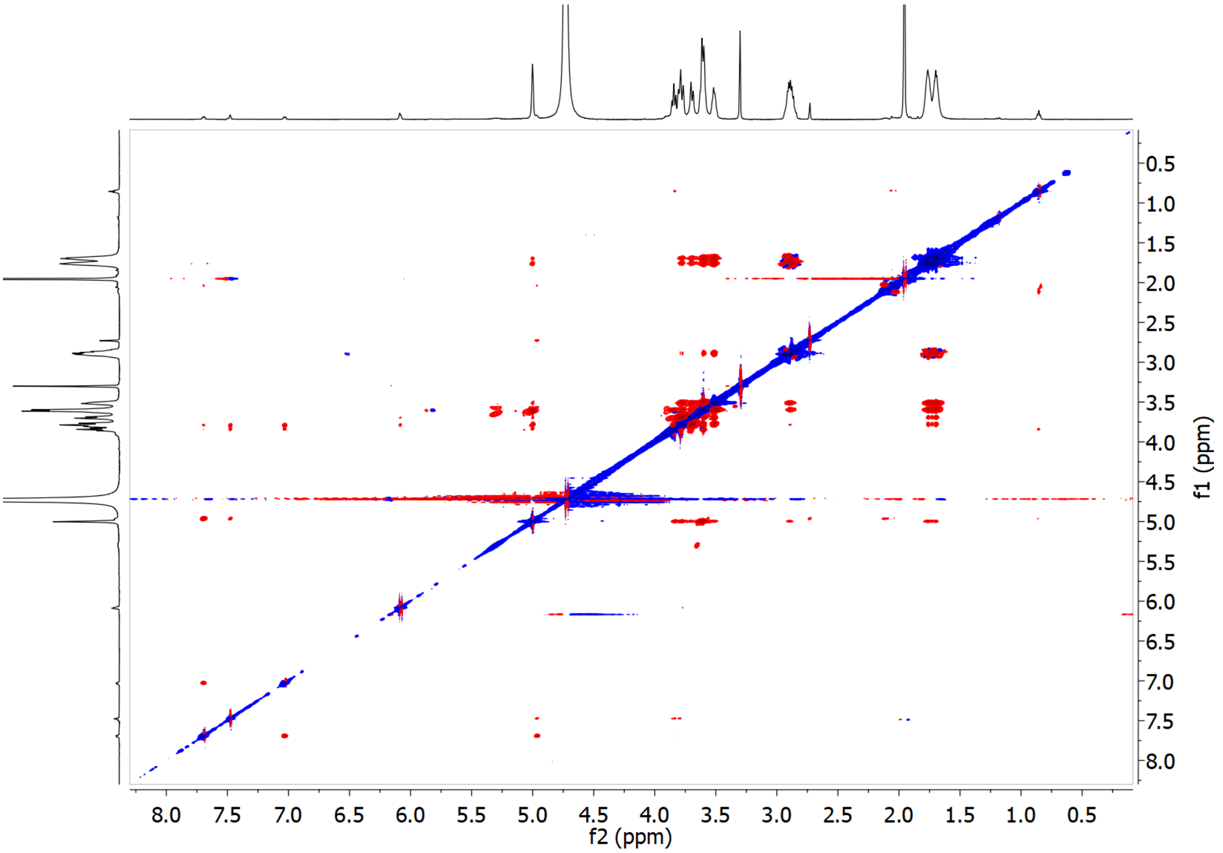

Figure S12. The 2D ROESY spectrum of butylone - Succ-β-CD complex. Further conditions can be found in *3.3. NMR experiments* section.

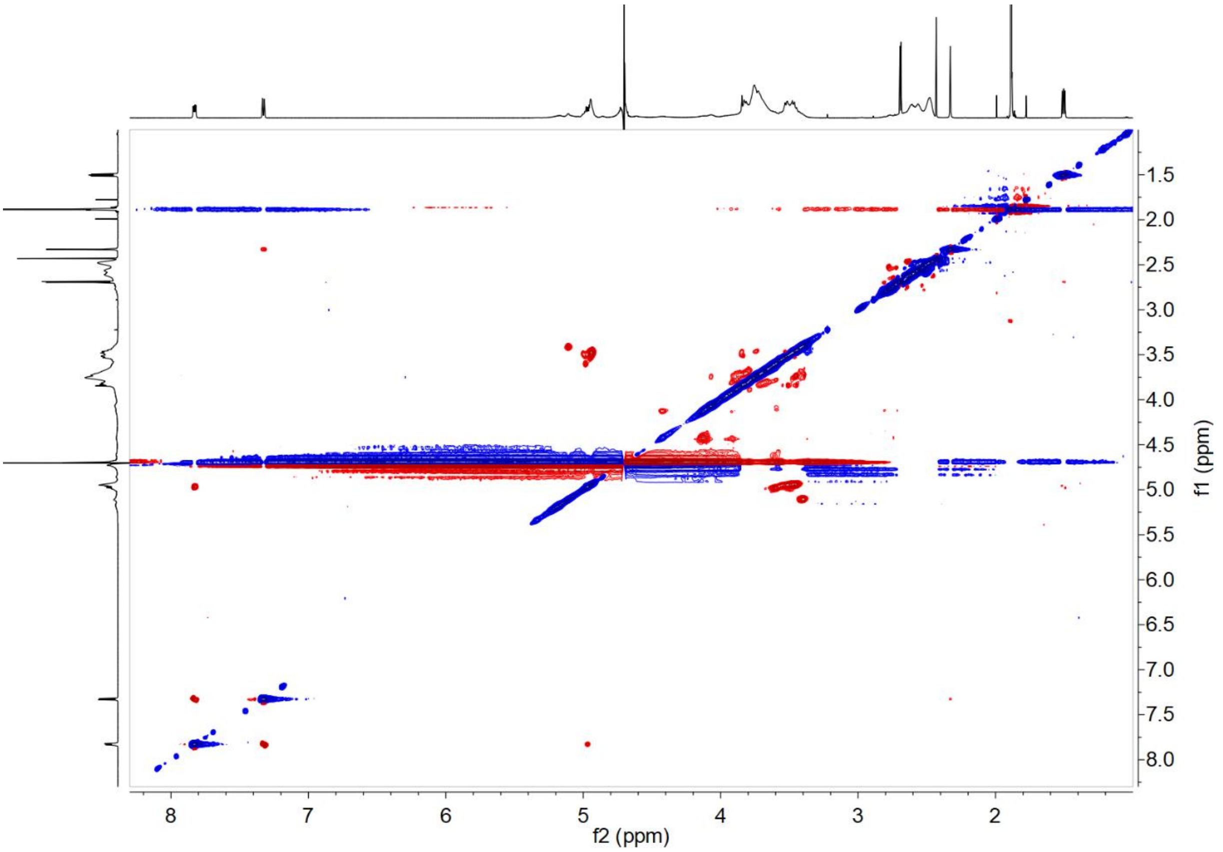

Figure S13. The 2D ROESY spectrum of mephedrone - Succ-β-CD complex. Further conditions can be found in *3.3. NMR experiments* section.

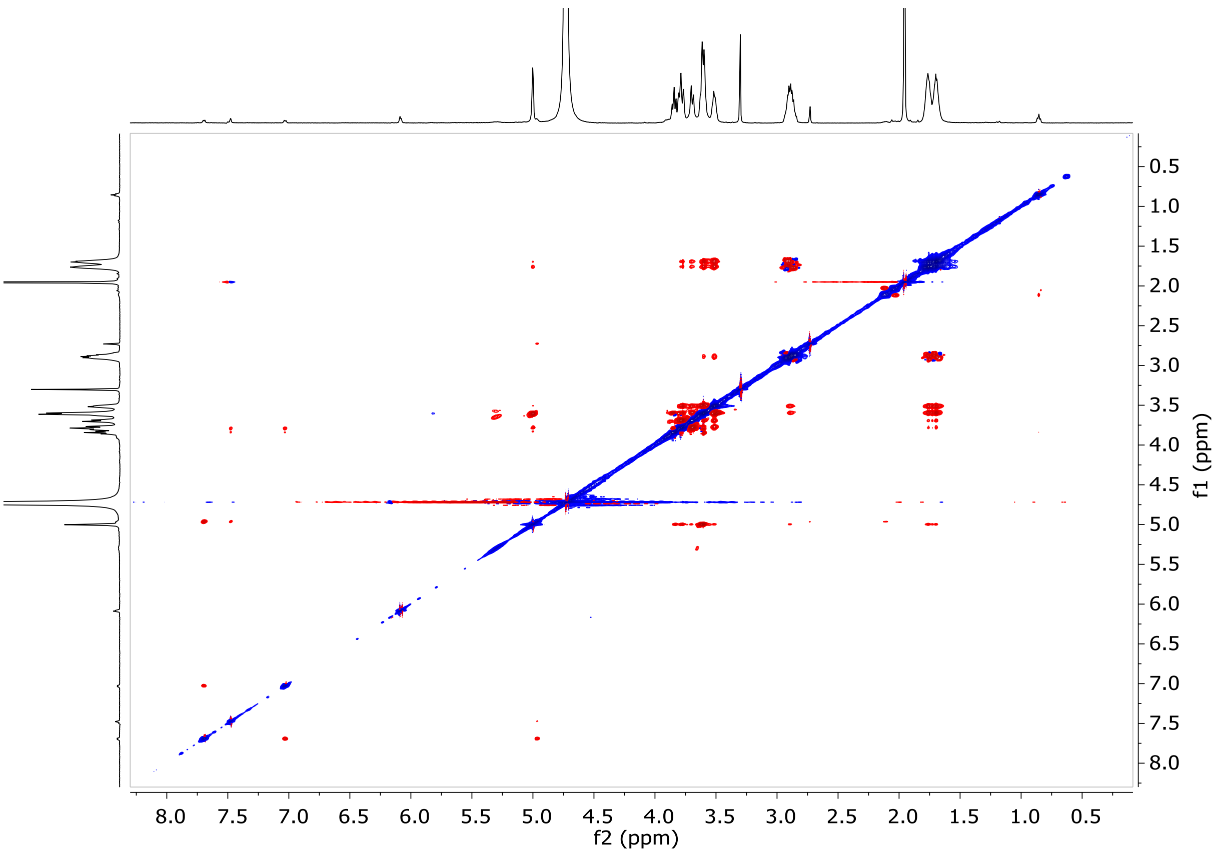

Figure S14. The 2D ROESY spectrum of butylone - 6-(SB)7-β-CD complex. Further conditions can be found in *3.3. NMR experiments* section.

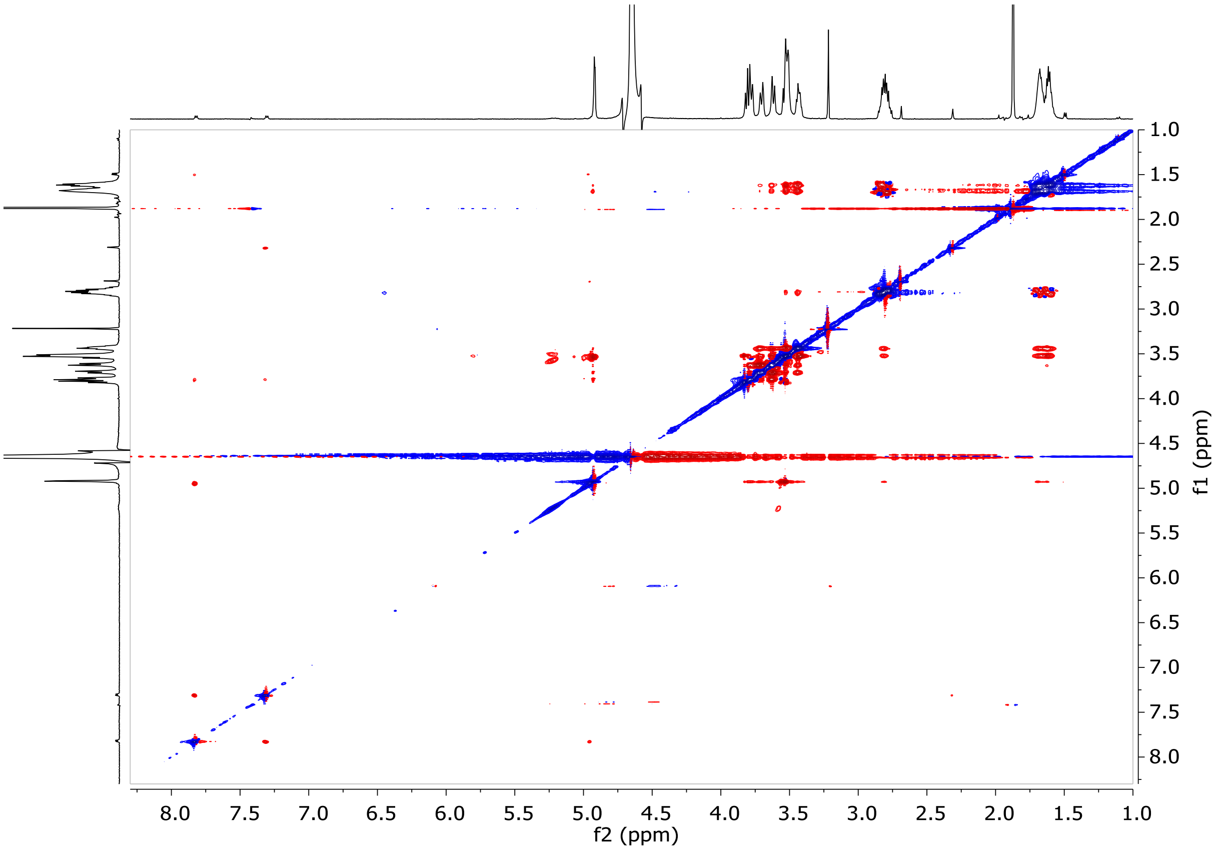

Figure S15. The 2D ROESY spectrum of mephedrone - 6-(SB)7-β-CD complex. Further conditions can be found in *3.3. NMR experiments* section.

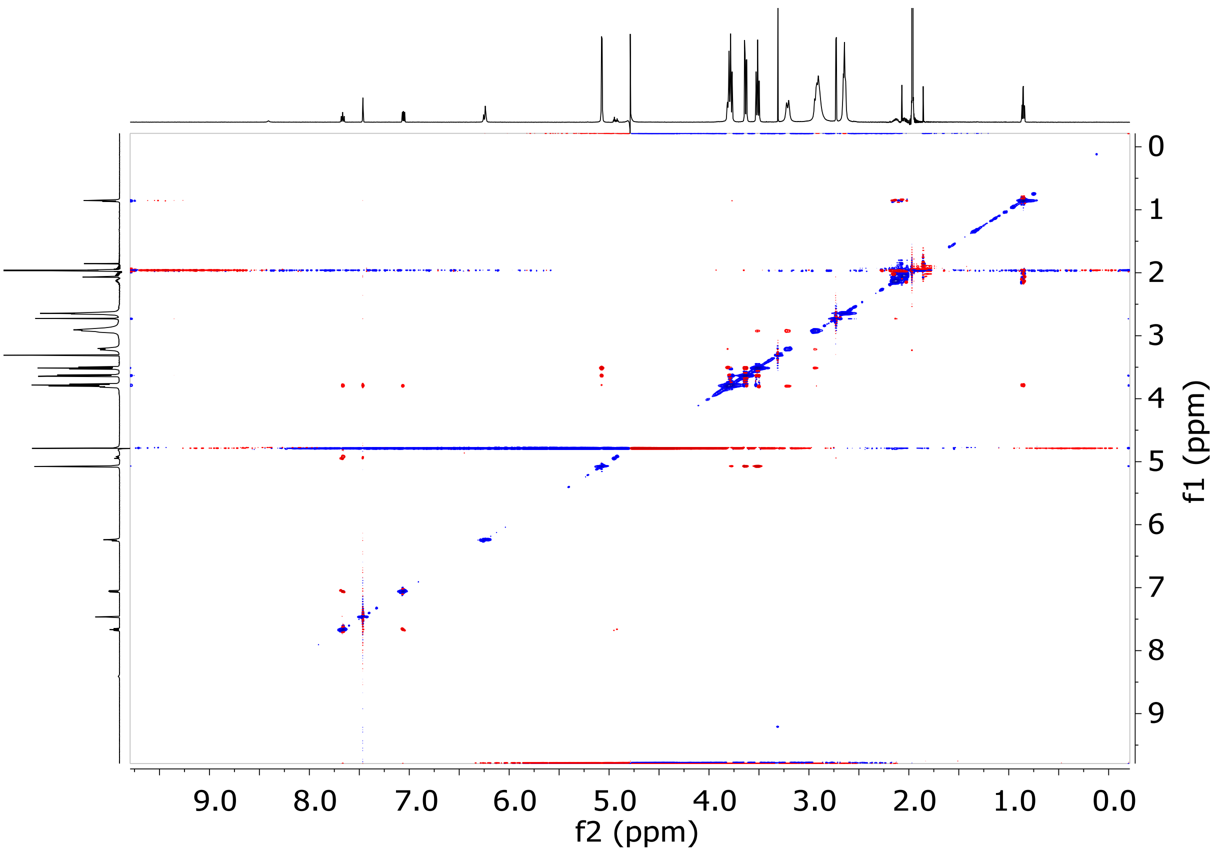

Figure S16. The 2D ROESY spectrum of butylone - SBX complex. Further conditions can be found in *3.3. NMR experiments* section.

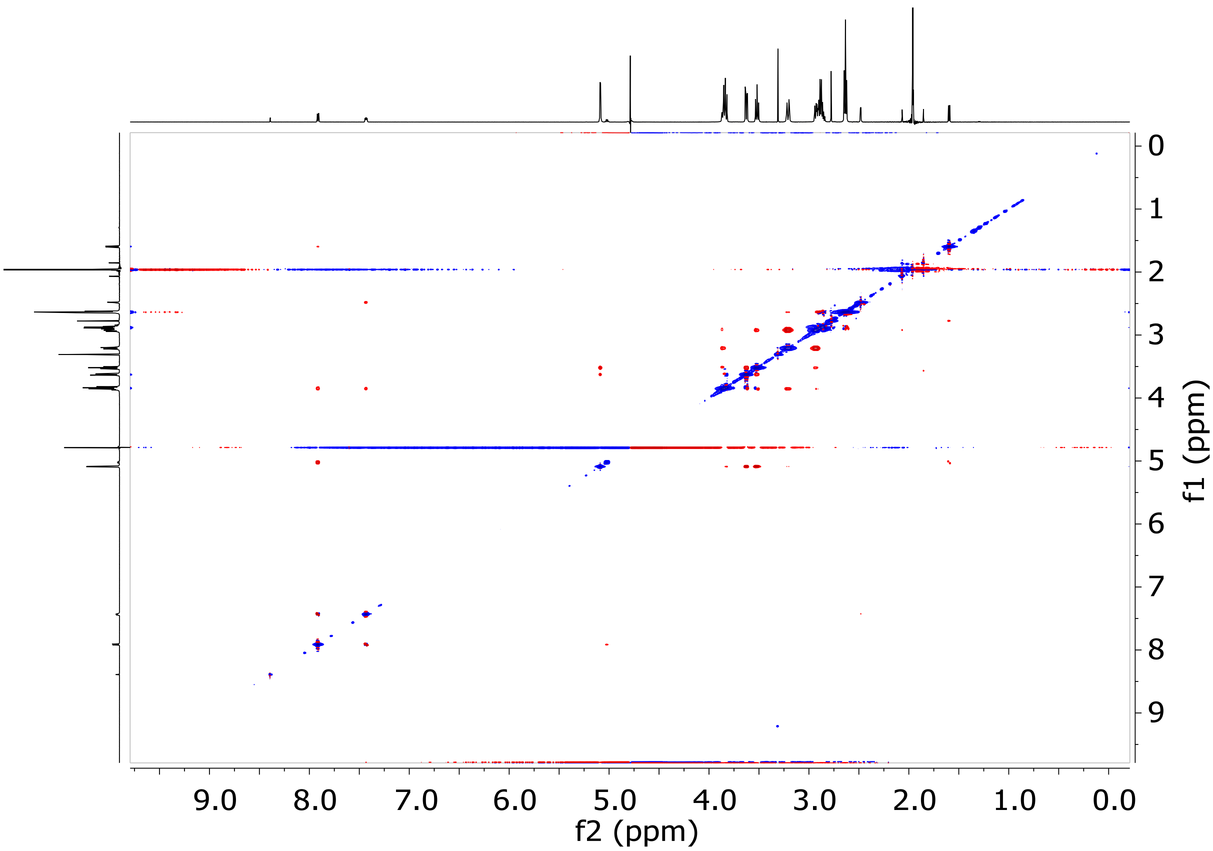

Figure S17. The 2D ROESY spectrum of mephedrone - SBX complex. Further conditions can be found in *3.3. NMR experiments* section.

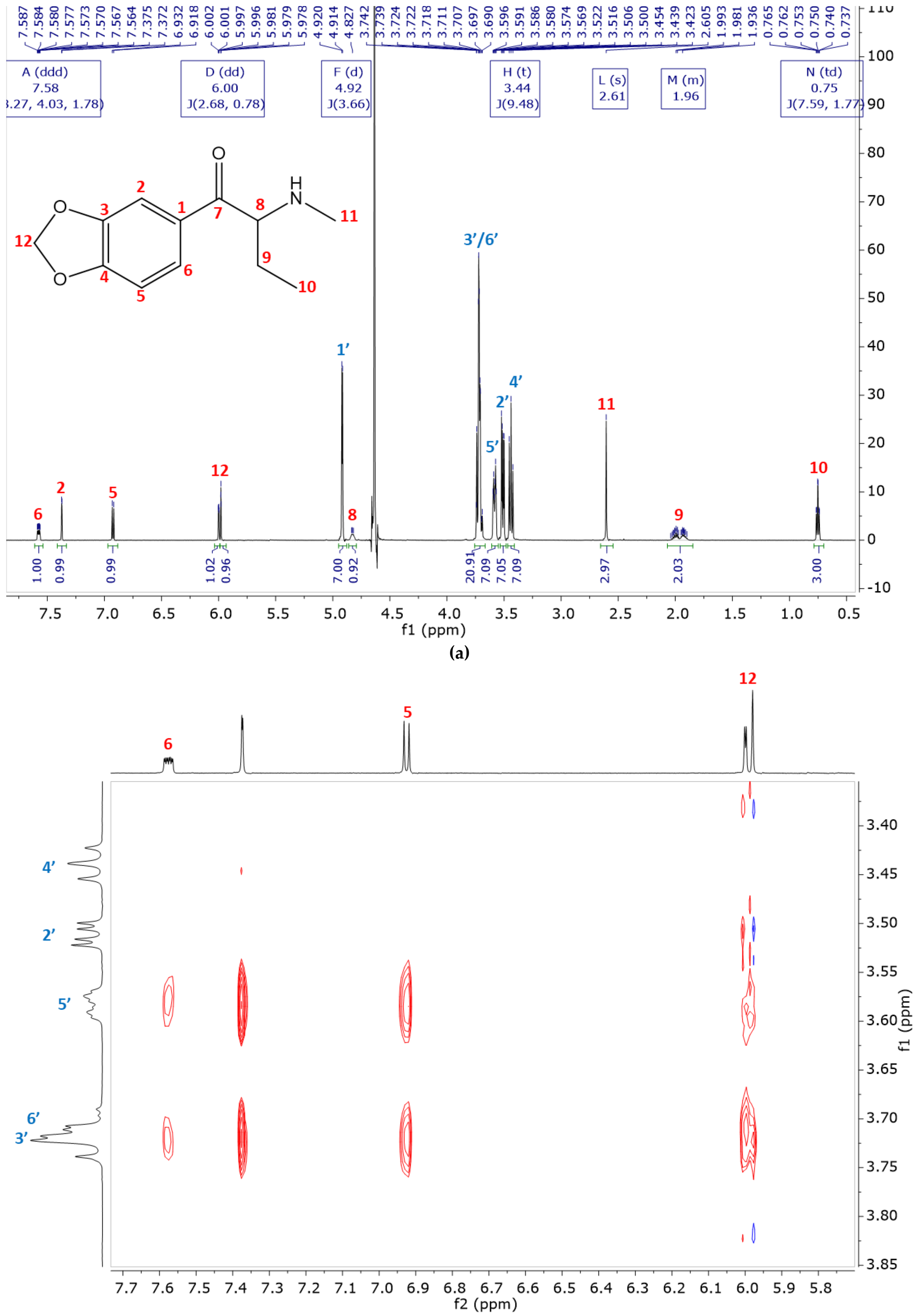

(a)

(b)

Figure S18. The ^{1}H NMR spectrum **(a)** and partial 2D ROESY spectrum **(b)** of butylone - β-CD complex. Further conditions can be found in *3.3. NMR experiments* section.

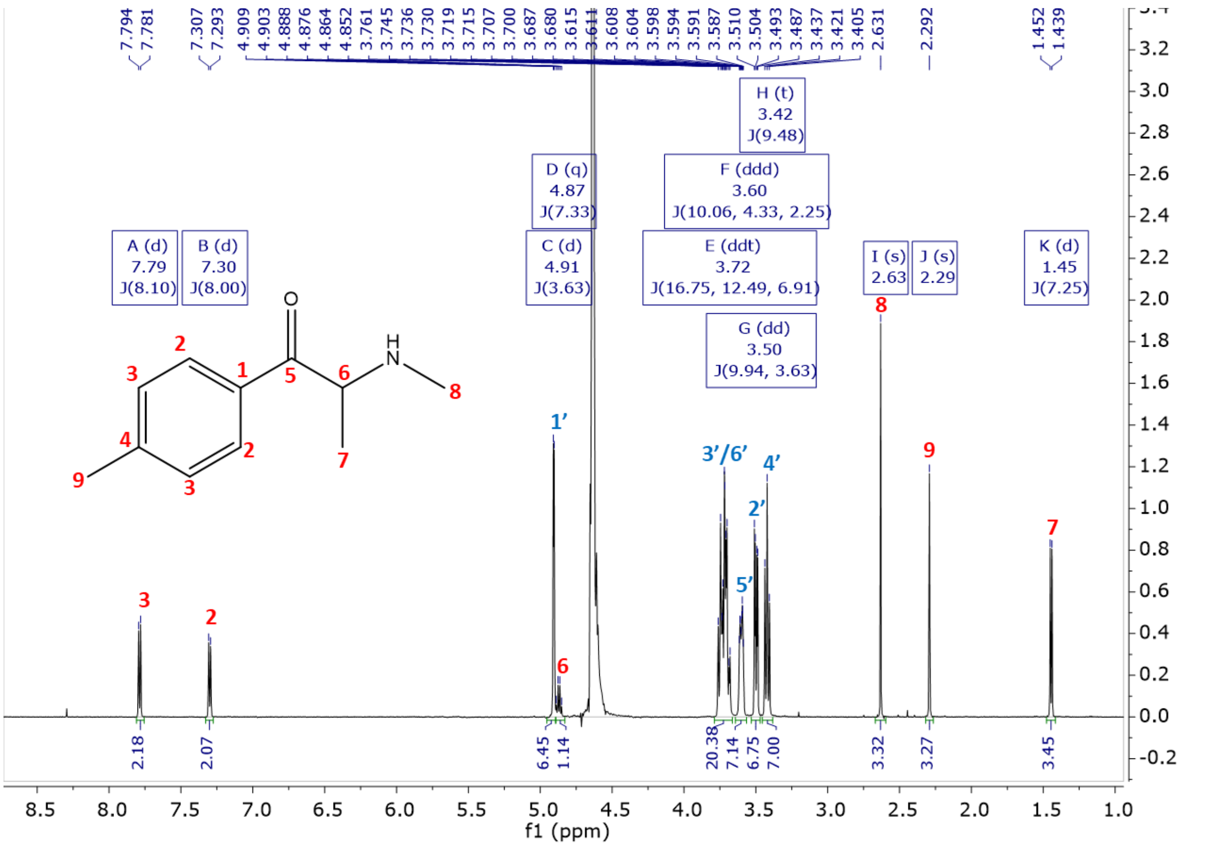

(a)

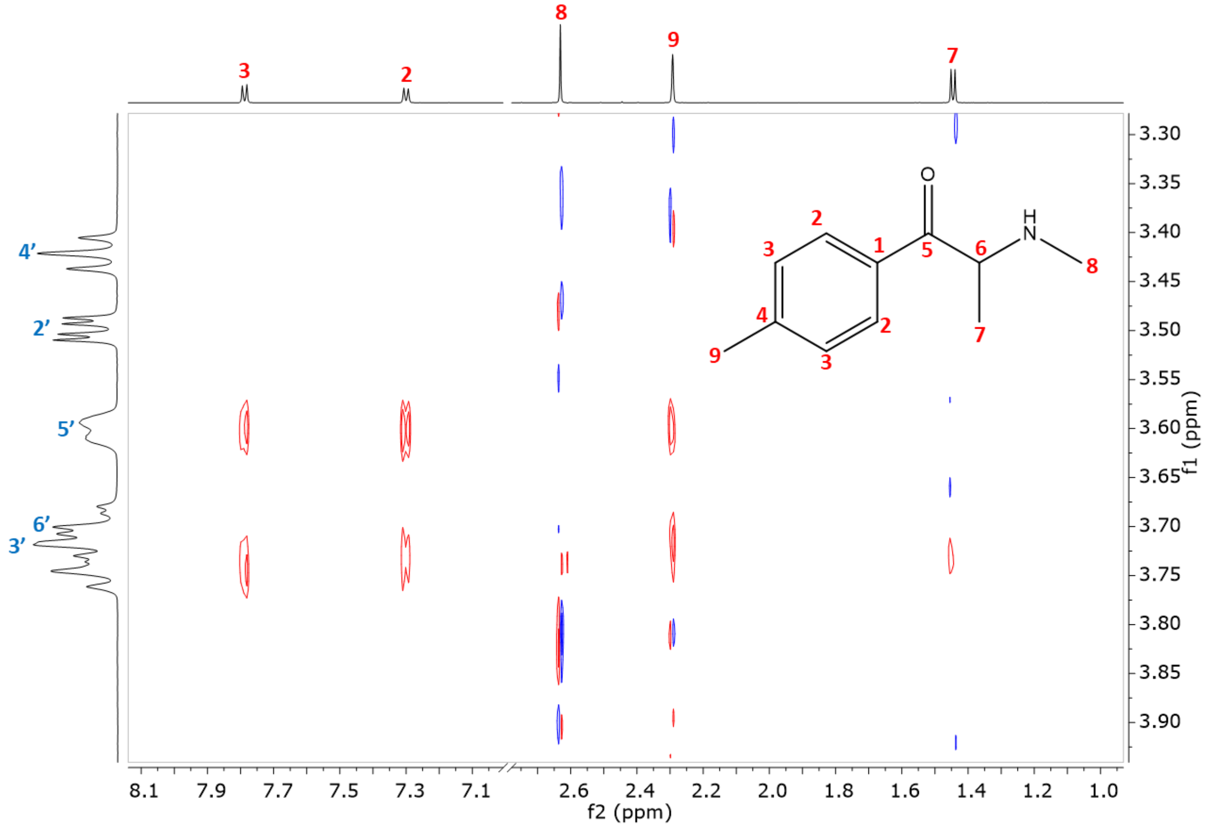

(b)

Figure S19. The ^{1}H NMR spectrum **(a)** and partial 2D ROESY spectrum **(b)** of mephedrone - β-CD complex. Further conditions can be found in *3.3. NMR experiments* section.

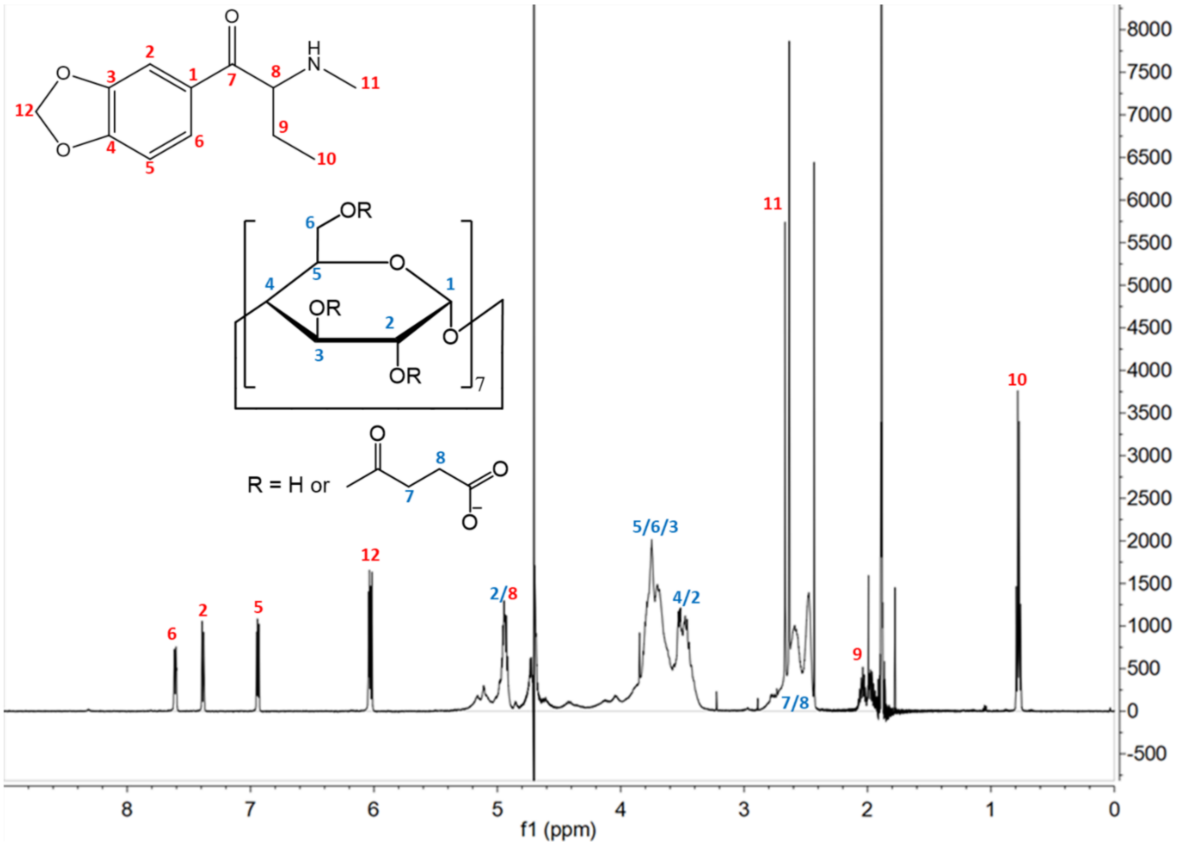

(a)

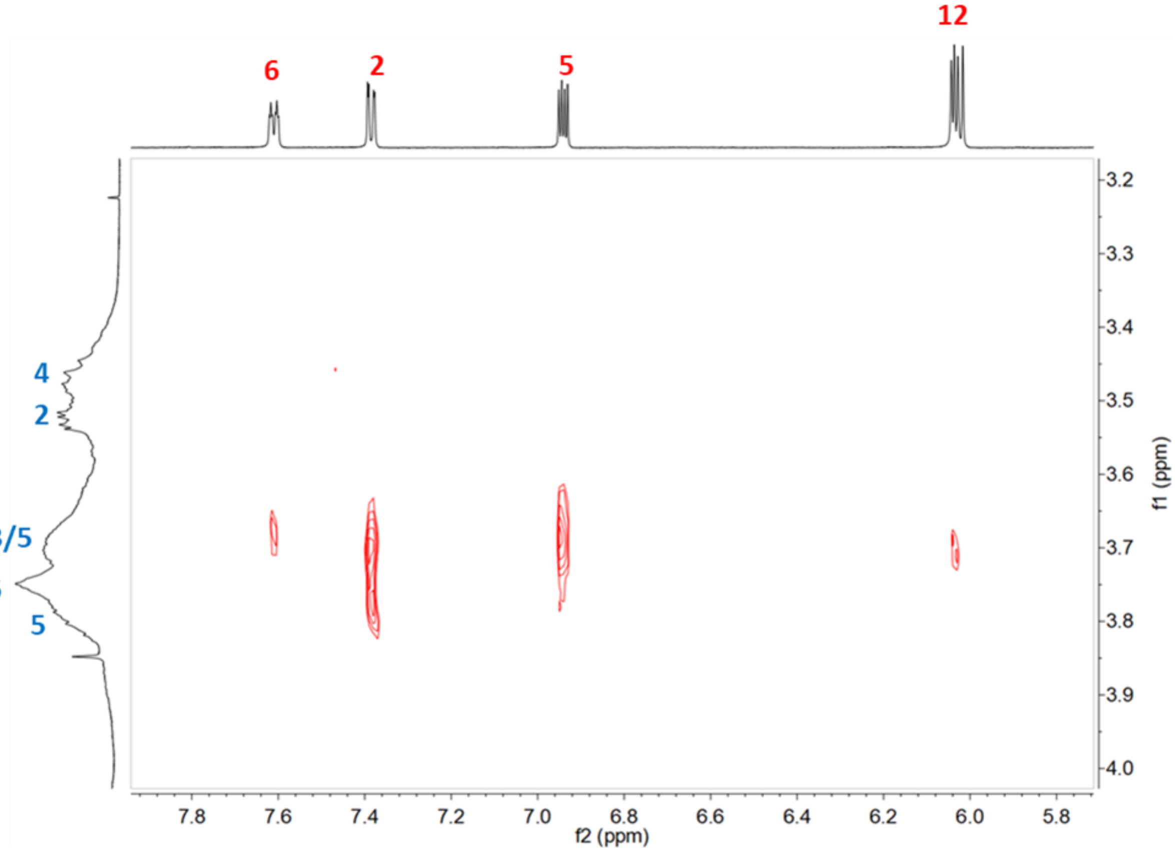

(b)

Figure S20. The ^{1}H NMR spectrum **(a)** and partial 2D ROESY spectrum **(b)** of butylone - Succ-β-CD complex. Further conditions can be found in *3.3. NMR experiments* section.

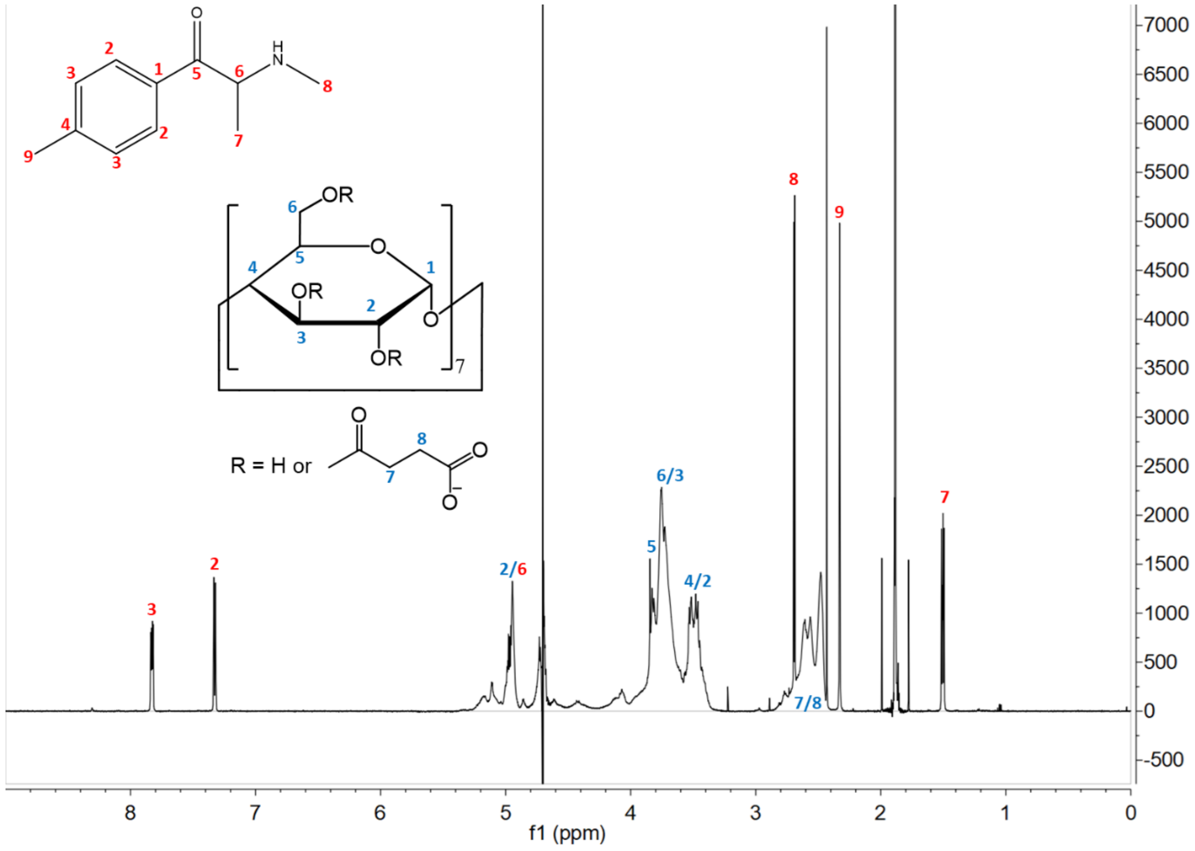

(a)

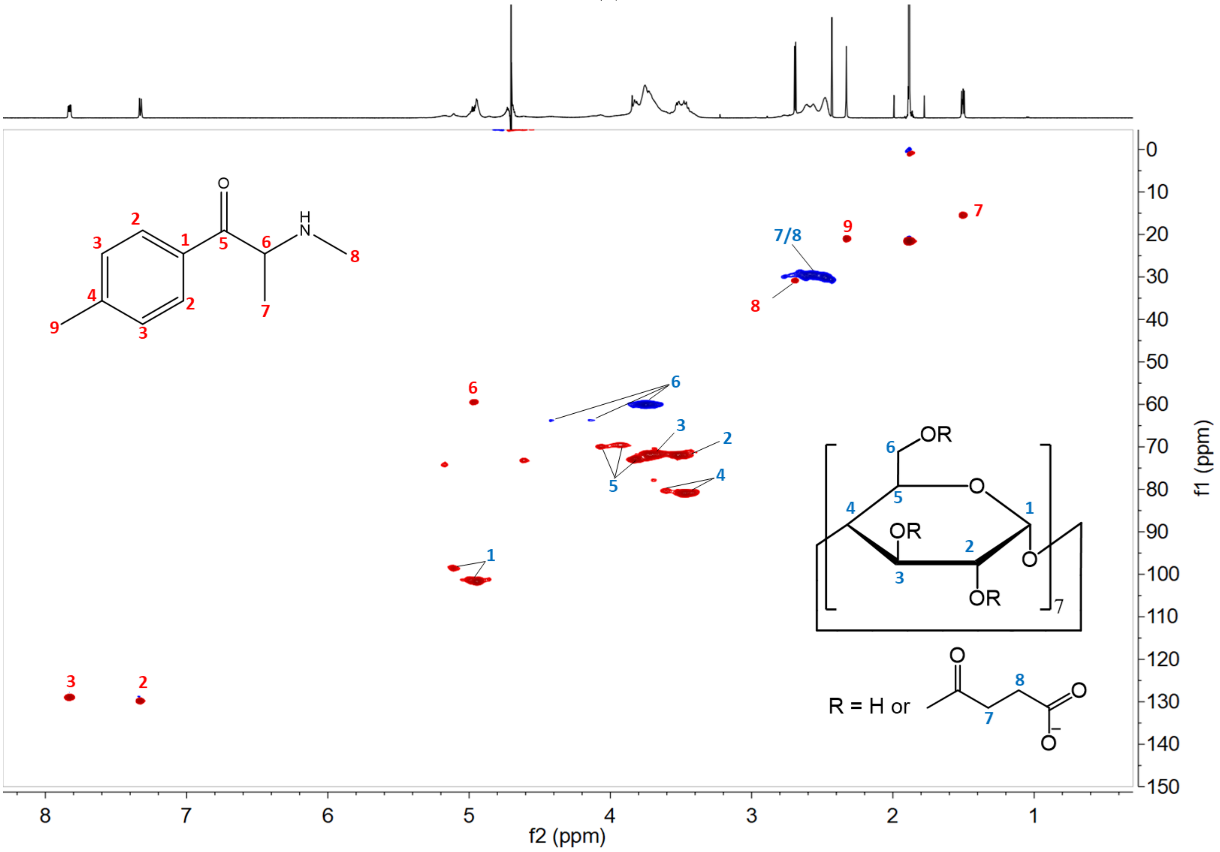

(b)

Figure S21. The ^{1}H NMR spectrum **(a)** and ^{1}H-^{13}C HSQC spectrum **(b)** of mephedrone - Succ-β-CD complex. Further conditions can be found in *3.3. NMR experiments* section.

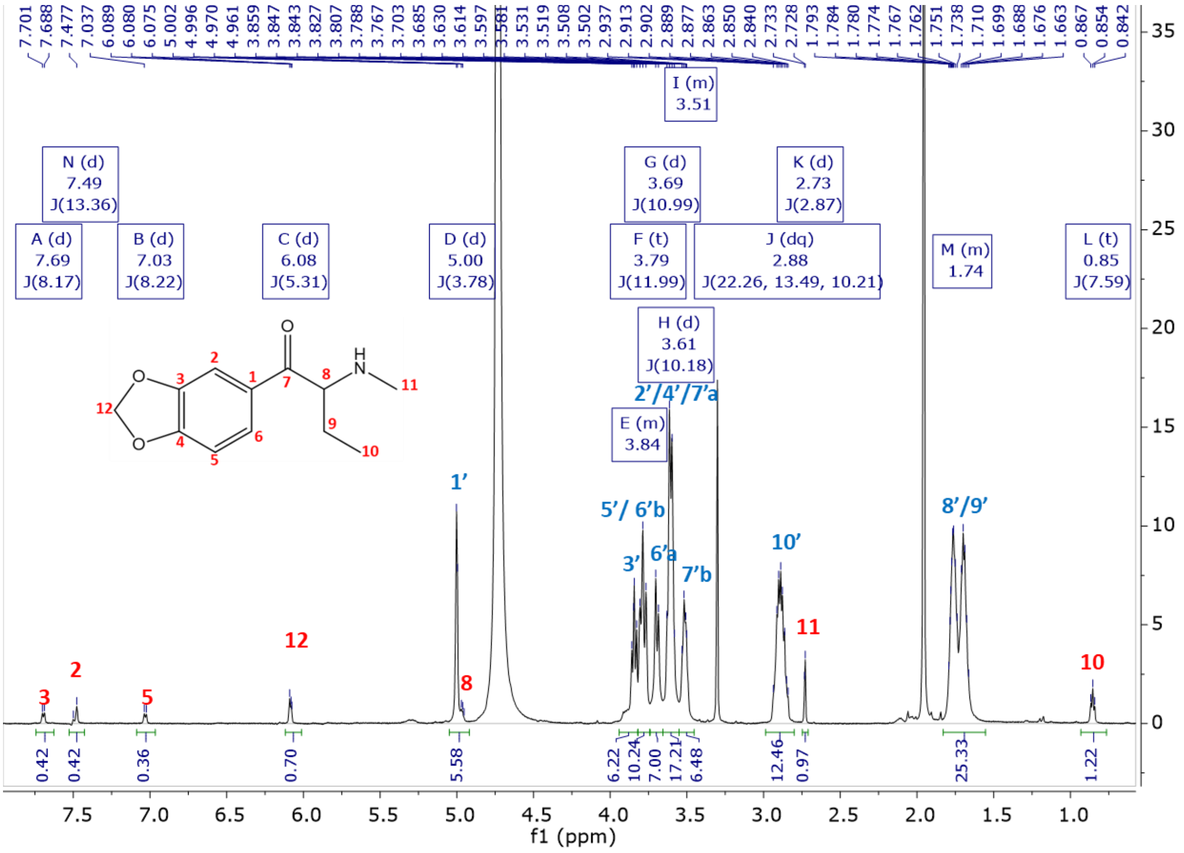

(a)

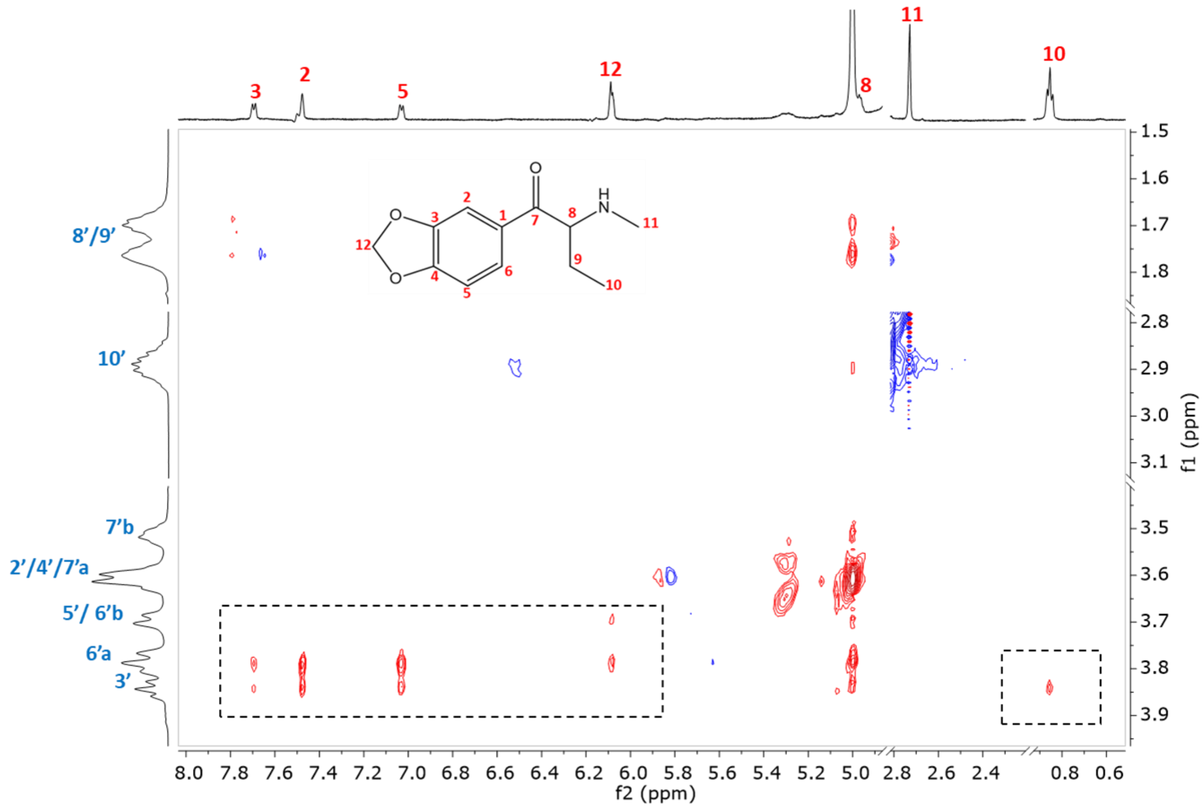

(b)

Figure S22. The ^{1}H NMR spectrum **(a)** and partial 2D ROESY spectrum **(b)** of butylone - 6-(SB)$_7$-β-CD complex. Further conditions can be found in *3.3. NMR experiments* section.

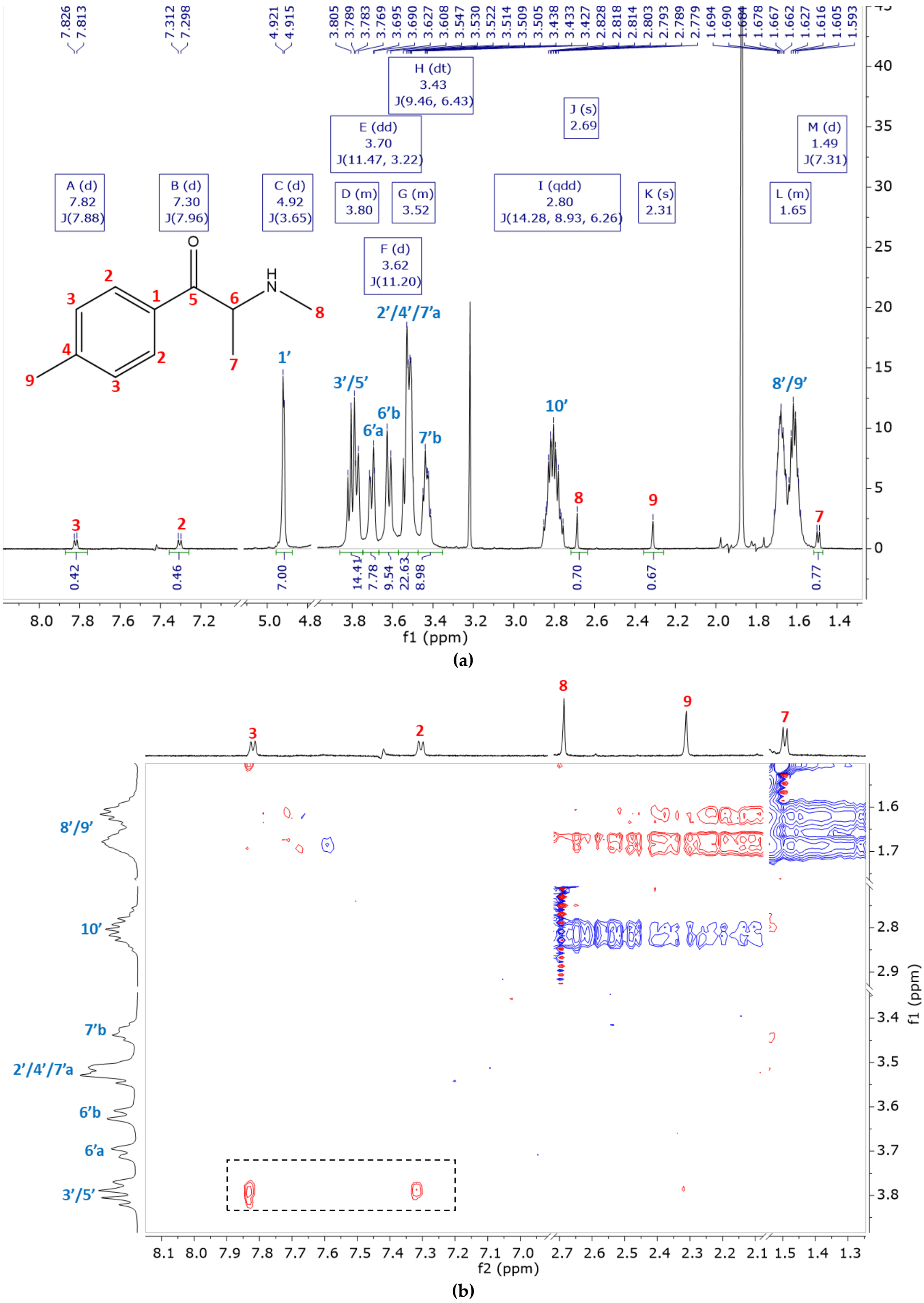

Figure S23. The ^{1}H NMR spectrum **(a)** and partial 2D ROESY spectrum **(b)** of mephedrone - 6-(SB)$_7$-β-CD complex. Further conditions can be found in *3.3. NMR experiments* section.

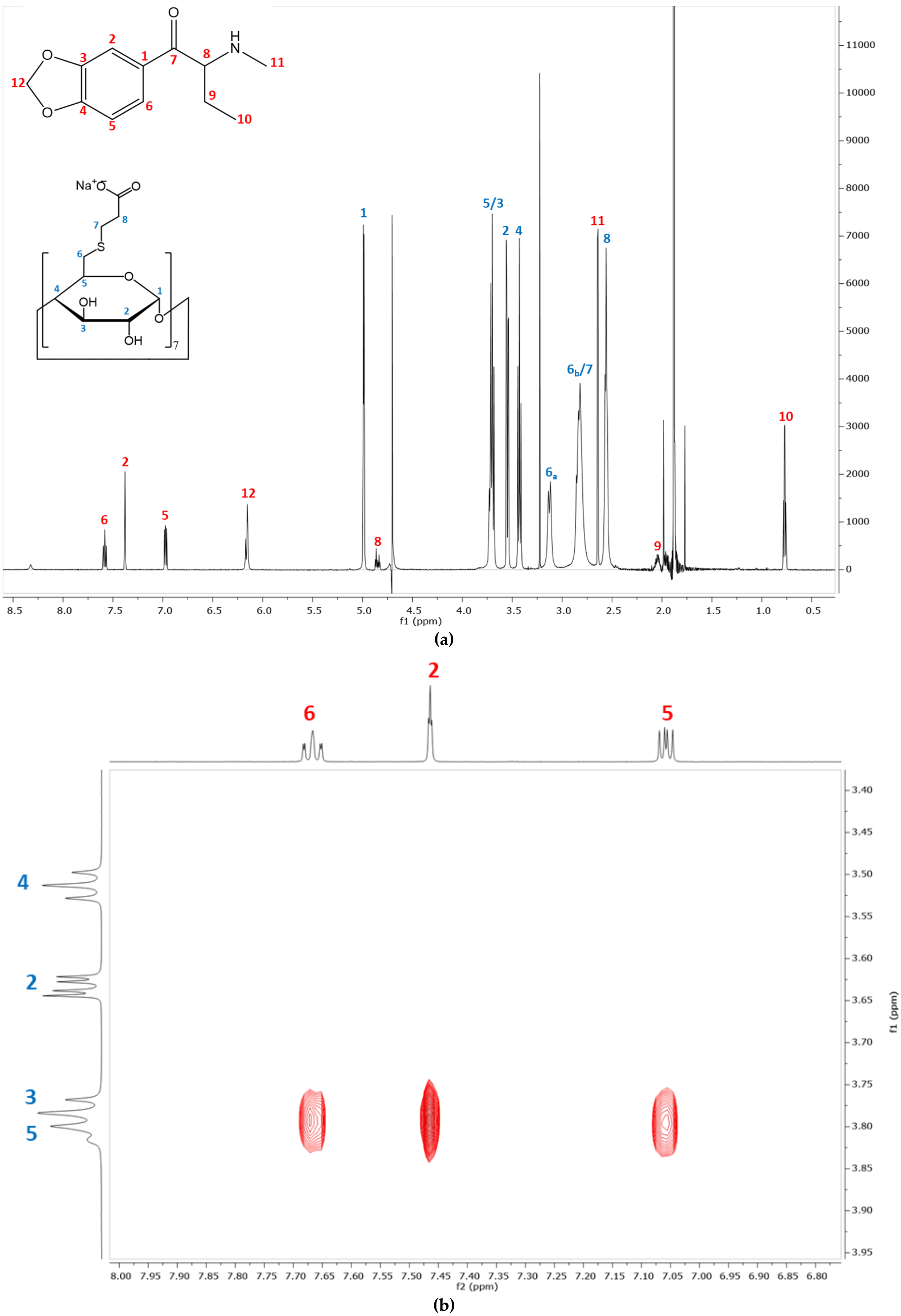

Figure S24. The ^{1}H NMR spectrum **(a)** and partial 2D ROESY spectrum **(b)** of butylone - SBX complex. Further conditions can be found in *3.3. NMR experiments* section.

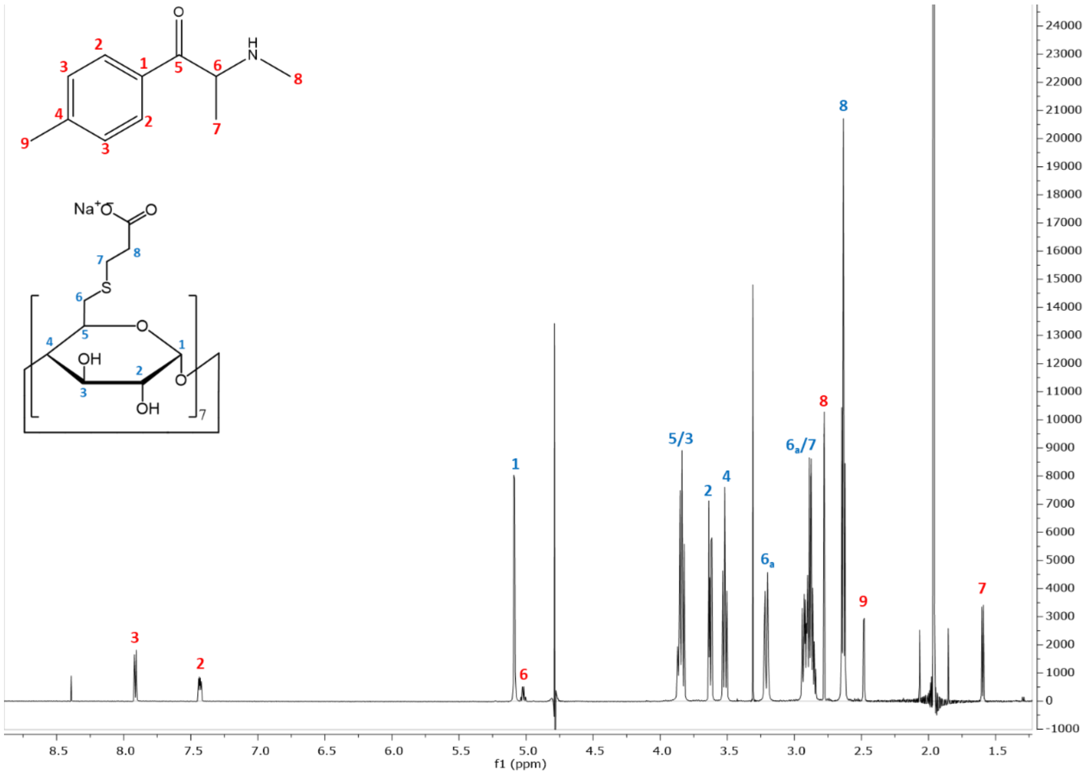

(a)

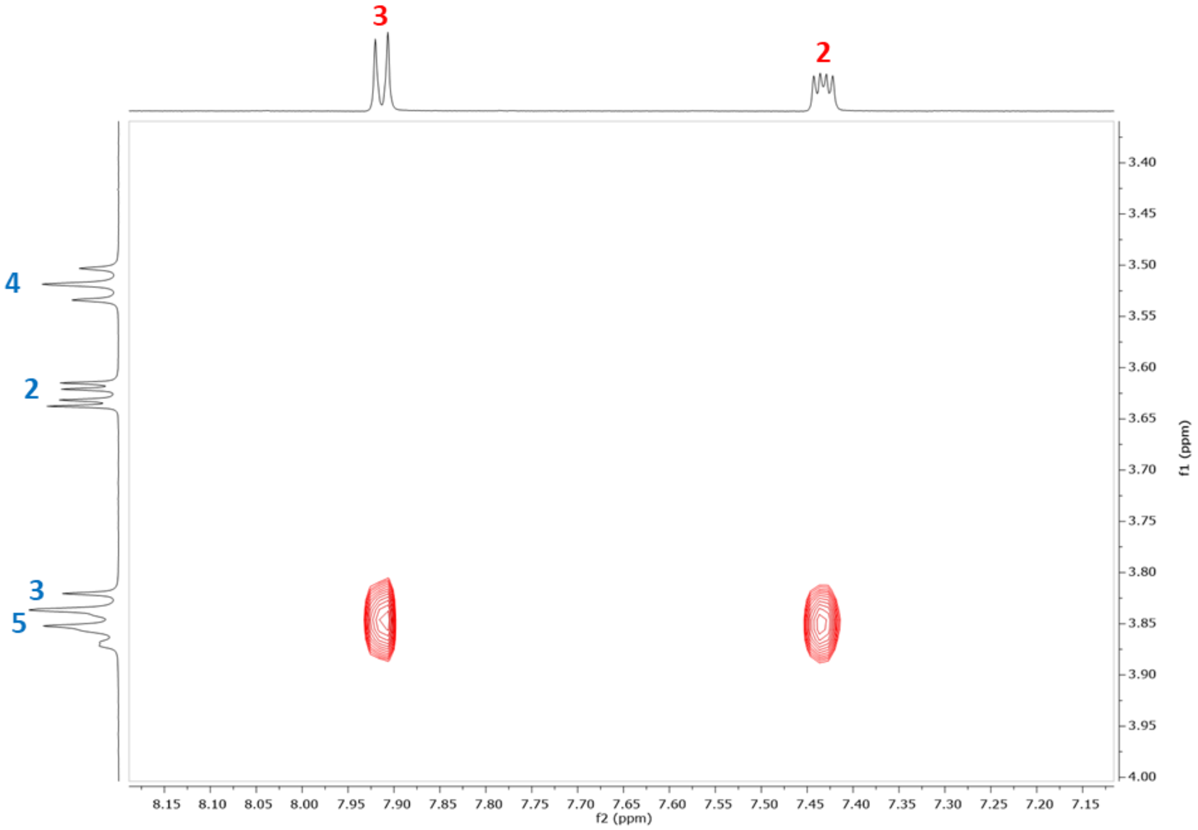

(b)

Figure S25. The ^{1}H NMR spectrum **(a)** and partial 2D ROESY spectrum **(b)** of mephedrone - SBX complex. Further conditions can be found in *3.3. NMR experiments* section.
